# ABCA1 transporter reduces amphotericin B cytotoxicity in mammalian cells

**DOI:** 10.1007/s00018-019-03154-w

**Published:** 2019-05-27

**Authors:** A. Wu, E. Grela, K. Wójtowicz, N. Filipczak, Y. Hamon, R. Luchowski, W. Grudziński, O. Raducka-Jaszul, M. Gagoś, A. Szczepaniak, G. Chimini, W. I. Gruszecki, T. Trombik

**Affiliations:** 1 Faculty of Biotechnology, University of Wroclaw, 50-383 Wrocław, Poland; 2 Department of Biophysics, Institute of Physics, Maria Curie-Sklodowska University, 20-031 Lublin, Poland; 3 Aix Marseille University, CNRS, INSERM, CIML, Marseille, France; 4 Department of Cell Biology, Maria Curie-Skłodowska University, 20-033 Lublin, Poland

**Keywords:** Plasma membrane, Raw 264.7 macrophages, CHO-K1, MTT cytotoxicity assays, FLIM, Fluorescence anisotropy

## Abstract

Amphotericin B (AmB) belongs to a group of polyene antibiotics commonly used in the treatment of systemic mycotic infections. A widely accepted mechanism of action of AmB is based on the formation of an oligomeric pore structure within the plasma membrane (PM) by interaction with membrane sterols. Although AmB binds preferentially to ergosterol, it can also bind to cholesterol in the mammalian PM and cause severe cellular toxicity. The lipid content and its lateral organization at the cell PM appear to be significant for AmB binding. Several ATP-binding cassette (ABC) transporters, including ABCA1, play a crucial role in lipid translocation, cholesterol redistribution and efflux. Here, we demonstrate that cells expressing ABCA1 are more resistant to AmB treatment, while cells lacking ABCA1 expression or expressing non-active ABCA1MM mutant display increased sensitivity. Further, a FLIM analysis of AmB-treated cells reveals a fraction of the antibiotic molecules, characterized by relatively high fluorescence lifetimes (> 6 ns), involved in formation of bulk cholesterol–AmB structures at the surface of ABCA1-expressing cells. Finally, lowering the cellular cholesterol content abolishes resistance of ABCA1-expressing cells to AmB. Therefore, we propose that ABCA1-mediated cholesterol efflux from cells induces formation of bulk cholesterol–AmB structures at the cell surface, preventing AmB cytotoxicity.

**Electronic supplementary material:**

The online version of this article (10.1007/s00018-019-03154-w) contains supplementary material, which is available to authorized users.

## Introduction

Amphotericin B (AmB) belongs to a group of lifesaving polyene antibiotics used in the treatment of systemic mycoses, whose number is growing, especially in immunosuppressed patients, and as a response to more and more widely used treatments with broad-spectrum antibacterial antibiotics. The principal and commonly accepted mechanism of action of this antibiotic is based on the formation of an oligomeric pore structure within the fungal plasma membrane (PM) by interaction with membrane sterols. This leads to the flux of cations, cell membrane depolarization and cell death [1–3]. Unfortunately, owing to operation of very similar mechanisms in human cells, AmB is also highly toxic to patients. Revealing the molecular mechanisms governing biological activity of AmB may lead to the development of a pharmacological formula of the drug characterized by potent antifungal activity with minimized toxicity to patients.

AmB demonstrates a very high tendency to form molecular aggregates, such as dimers and higher dimension supramolecular structures, in all environments important from the physiological point of view, including the water phase and lipid membranes. Importantly, the sterols present in biomembranes, both cholesterol present in human cells and ergosterol present in fungi, modulate molecular organization of AmB and binding of the drug into membranes. On the one hand, the presence of sterols in the lipid phase is a factor that allows the drug to be incorporated into the membrane (intramembranous mode) [4–6]. On the other hand, AmB has been demonstrated to effectively “pull out” sterol molecules from the lipid phase and immobilize them within extramembranous, sterol–AmB hybrid structures [7]. Both mechanisms are relevant from the standpoint of the mode of action of AmB. The first one leads to formation of pore-like, transmembrane structures disrupting physiological ion transport, and the second one can affect structural and dynamic properties of biomembranes. NMR studies show that the presence of ergosterol significantly affects AmB mobility in lipid bilayers [8]. Sterol-rich nanodomains or lipid rafts appear to be an important feature leading to AmB binding and toxicity. In contrast, in sterol-free, liquid-disordered (Ld) membranes, AmB has low orientational and positional stability [6, 9]. This suggests that the lipid-ordered phase in model membrane (considered as experimental lipid rafts) favors the formation of stable channels [5].

Lipids and sterols are essential components of the cell PM, whose arrangements determine the physicochemical properties of cell membranes, tuning intracellular signaling and whole cell homeostasis. The plasma membrane compartmentalization is characterized by the presence of extremely dynamic membrane nanodomains (tens to hundreds of nm in size), which are in constant motion. These structures can have different chemical natures (lipid, protein, protein–lipid), origin and function, and their presence at the PM is tightly coordinated. Although huge progress has been made in the analysis of membrane domains, mainly by biophysical techniques, the determinants dictating local organization of the PM remain poorly understood. Nevertheless, the interactions between proteins and lipids seem to be crucial in the spatial distribution and chemical modification of membrane nanodomains (for review see: [10–14]). In this context, several ATP-binding cassette (ABC) transporters exert a crucial role in lipid translocation, orchestrating cholesterol organization, redistribution and efflux. Among them, ABCA1, the prototype of the ABCA subfamily, has been shown to promote high-density lipoprotein (HDL) formation, cellular cholesterol efflux and reverse cholesterol transport (RCT) from the peripheral tissues to the liver (for review see: [15–20]), as a central player in the metabolism of cellular lipids and cholesterol. ABCA1 loss-of-function leads to the development of the genetic disorder Tangier disease, in which impaired reverse cholesterol transport and accumulation of lipids have been observed [21]. Although the 3D structure of this transporter has been recently resolved [22], the mechanism of interaction between transporter, apolipoproteins and subsequent lipid and cholesterol efflux is still being discussed. It has been reported that ABCA1 elicits an outward flip of negatively charged phosphatidylserine at the PM [23, 24]. In addition, fluorescence lifetime imaging microscopy (FLIM) experiments have provided the first evidence that ABCA1 increases the lipid packing of the exoplasmic leaflet, and redistributes cholesterol present in the lipid raft between smaller pools [25]. It was later confirmed that nascent HDL molecules originate from PM lipid rafts under the coordination of ABCA1 [26]. Moreover, it has been shown that the expression of ABCA1 correlates with upregulation of the liver X receptor (LXR), causing inhibition of MAPK signaling and cell division in some cancer cells. This is probably due to the reorganization and efflux of cholesterol pools at the PM [27, 28]. Thus, ABCA1 appears as a key controller of the lipid microenvironments with direct effects on the general molecular organization of the PM and subsequent processes. We thus hypothesized a role for ABCA1 in AmB interactions with the cell plasma membrane.

Despite significant progress in the field, particularly regarding model membranes, the molecular mechanisms of AmB selectivity and reactivity with fungal and mammalian cell membranes in vivo remain poorly understood. Revealing these mechanisms would not only broaden the current knowledge, but more importantly could help in the development of less toxic AmB-based therapies. In the present study, we propose a novel mechanism of cell resistance against AmB, in which active efflux of cholesterol mediated by the ABCA1 transporter provokes formation of bulk cholesterol–AmB structures at the cell surface, preventing AmB from penetrating the cell plasma membrane and causing cytotoxicity.

## Materials and methods

### Cloning of *Pgk* promoter

Original pBudCE4.1 plasmids (Invitrogen) containing a gene encoding the wild-type or MM mutant of ABCA1 transporter fused with eGFP under the control of the *Ef1α* promoter were digested by *Xba*I restrictive enzyme. This digestion removed a fragment containing the Zeocin resistance gene (*Zeo*^*r*^) and *Ef1α* promoter upstream of the *Abca1* gene. Next, the *Zeo*^*r*^ gene was amplified by PCR using the following primers: *Zeo_F* (5′ATCGATCTTAAGCAGTACTTCTAGAGGACT3′) and *Zeo_R* (5′GCGCCTCCCCTACCCGGTAGGAAGCTAGCTCGACGAGGGTG3′) on the matrix of pBudCE4.1, and the mouse *Pgk* promoter [29] was amplified by PCR using the following primers: *pPGK_F* (5′ACCCTCGTCGAGCTAGCTTCCTACCGGGTAGGGGAGGCGC3′) and *pPGK_R* (5′GGGGGATCCACTAGTTCTAGAGCGGCCGCGACCACGTGTCGAAAGGCCCGGAGATGAGG3′) on the matrix of MXS_PGK vector [30]. Finally, both PCR fragments containing *Zeo*^*r*^ and the mouse *Pgk* promoter were ligated with the *Xba*I linearized vector containing the *Abca1* or *abca1mm* gene using the Gibson assembly kit (New England Biolabs). After subcloning in *E. coli* DH5α, the new plasmids were verified by sequencing and used for CHO-K1 cell transfection.

### Cells

CHO-K1 (RCB0285, Riken Cell Bank) cells were cultured in Ham’s F-12 Nutrient Mix (Gibco) supplemented with 10% new born calf serum (NBCS, Gibco), 100 U/mL penicillin (Gibco), 100 µg/mL streptomycin (Gibco) and 2 mM l-glutamine (Gibco) (complete Ham’s F12 medium). Raw 264.7 macrophages (91062702, ATCC) were cultured in Dulbecco’s Modified Eagle’s Medium (DMEM, Gibco) supplemented with 10% fetal bovine serum (FBS, Gibco), 100 U/mL penicillin, 100 µg/mL streptomycin and 2 mM l-glutamine (complete DMEM medium). All cells were cultured at 37 °C in a humidified atmosphere containing 5% CO_2_. CHO-K1 cells were transfected using Lipofectamine 3000 (Life-Technologies). After transfection and selection in the presence of Zeocin (150 µg/mL), a few clones for each plasmid emerged. These clones were isolated and cultured, and each clone was verified by flow cytometry (FACS) regarding GFP expression. One clone of each, stably expressing either ABCA1-GFP (A1G) or ABCA1MM-GFP (MMG), was used in this work. Selected A1G and MMG clonal lines were routinely cultured in the complete Ham’s F12 medium supplemented with 100 µg/mL of Zeocin. ABCA1 expression in Raw 264.7 macrophages was induced by incubation of cells with 1 µM GW3965 in complete DMEM medium for 24 h prior to the experiment. Rat hybridoma cells (clone 3A1-891.3 and 5A1-1422) were cultured in complete DMEM medium containing 7.5% ultra-low IgG FBS (VWR Life Science Seradigm) until the total culture volume reached approximately 150 mL. Afterwards, the culture was continued with progressive increase of the volume and decrease of FBS concentration until it dropped to less than 1%. At the end, the cells were maintained in these culture conditions for an additional 7 days. Finally, the cells were harvested and the cell culture medium containing antibodies was filtered through a 0.22-µm filter and kept for antibody purification. All cells were cultured in *Mycoplasma*-free conditions. The ABCA1 expression level was routinely controlled by flow cytometry.

### Antibodies and reagents

Monoclonal rat antibodies directed against mouse ABCA1 (clone 3A1-891.3 and 5A1-1422) were purified from hybridoma culture media by affinity chromatography on Protein G agarose (Merck Millipore) using the ÄKTA pure chromatography system (GE). Antibody against ABCG1 (NB400-132SS) was purchased from Novus Biologicals. Anti-rabbit IgG HRP (Ref. 31466) and anti-Rat IgG HRP (Ref. A18739) were purchased from Life Technologies. GW 3965, amphotericin B (AmB), probucol, zaragozic acid (ZA), dithiothreitol (DTT), protease and phosphatase inhibitor cocktails were purchased from Sigma-Aldrich. Bovine serum albumin (BSA) and dimethyl sulfoxide were purchased from Bioshop. Pierce Universal Nuclease for Cell Lysis for protein extraction was purchased from Thermo Fisher Scientific. Cholesterol was purchased from Northern Lipids Inc. and methyl-β-cyclodextrin (MβCD) from Alfa Aesar. All commonly used biochemical reagents stated below were purchased either from Sigma-Aldrich or Bioshop. Molecular biology reagents including restriction enzymes, Q5 polymerase, and Gibson Assembly kit were purchased from New England Biolabs.

Anti-ABCA1 antibody clone 5A1-1422 and apolipoprotein A-1 (ApoA1, Sigma-Aldrich) were coupled with Alexa Fluor 647 (Life Technologies) according to the manufacturer’s protocol. The excess of free Alexa dye was removed by size-exchange chromatography on Zeba columns (Thermo Fisher Scientific). The concentration of labeled protein and protein/dye ratio was measured on a NanoDrop 2000 spectrophotometer (Thermo Fisher Scientific).

### Western blot analysis

Equal amounts of cells (typically 5 × 10^6^ cells) were lysed in 200 µL of lysis buffer (4% CHAPS, 7 M urea, 2 M thiourea, 2 µg/mL nuclease and 1 × complete protease inhibitor cocktail) for 30 min on ice. Twenty microliters of two times concentrated loading buffer (8 M urea, 250 mM Tris/HCl pH 6.8, 10% SDS, 20% glycerol, 0.008% bromophenol blue, 100 mM DTT) was added to the 20 µL of cell lysate (equivalent of 5 × 10^5^ cells) and incubated at 37 °C for 10 min then centrifuged at 16,000*g* for 10 min. The supernatant was kept at 37 °C until loading. Proteins were separated by 5.5% sodium dodecyl sulfate polyacrylamide gel electrophoresis (SDS-PAGE) and electro-transferred to polyvinylidene fluoride (PVDF, GE) membranes using the Trans-Blot Turbo transfer system (Bio-Rad) in transfer buffer (48 mM Tris, 39 mM glycine, 0.1% SDS, 10% methanol, pH 9.2). To control the equal protein charge in each line, the PVDF membranes were stained with 0.2% Red Ponceau S solution (Sigma-Aldrich) and washed a few times with water. After blocking in 5% skimmed milk in TBS-T (50 mM Tris/HCl pH 7.6, 150 mM NaCl supplemented with 0.05% Tween-20) for 1 h at room temperature or overnight at 4 °C, membranes were incubated with primary antibodies (3 µg/mL for anti-ABCA1 clone 3A1-891.3 or 1 µg/mL for anti-ABCG1) in 1% skimmed milk in TBS-T overnight at 4 °C or for 3 h at room temperature. Excess of primary antibodies was removed by washing the membrane three times in 1% skimmed milk in TBS-T before incubation with horseradish peroxidase-labeled secondary antibody (0.1 μg/mL) for 1 h. After several washes with TBS-T, the presence of protein was revealed using Western Lightning Plus-ECL (PerkinElmer) on a ChemiDoc MP System with ImageLab software (Bio-Rad).

### Microscopy imaging

A1G and MMG cells were seeded at 1 × 10^4^ cells/well in Lab-Tek chambers (Nunc) in complete Ham’s F-12 medium and incubated for 48 h at 37 °C. Cells were then washed three times with HBSS (Gibco) supplemented with 10 mM HEPES, pH 7.4 (Gibco), and were imaged using a 63 × oil immersion objective on a LEICA SP8 confocal microscope (LEICA) with LAS X software.

### Flow cytometry

CHO-K1, A1G and MMG cells were harvested at 5 × 10^5^ cells/condition and washed twice with PBS buffer (Gibco) then analyzed on a NovoCyte Flow Cytometer (ACEA Biosciences) using NovoExpress software. Mock and GW3965-treated Raw 264.7 macrophages were harvested at 5 × 10^5^ cells/condition, washed twice with PBS buffer and blocked with PBS containing 2.5% BSA for 30 min at 4 °C. Cells were then labeled with 1 µg/mL of anti-ABCA1 coupled directly with Alexa Fluor-647 (1422-AF647) for 1 h at 4 °C, washed three times with PBS buffer and analyzed.

### ApoA1 binding

A1G and MMG cells were harvested at 3 × 10^5^ cells/condition and washed twice with a binding buffer (10 mM HEPES pH 7.4, 150 mM NaCl, 1.8 mM CaCl_2_, 5 mM KCl and 1 mM MgCl_2_). Cells were then resuspended in binding buffer supplemented with 0.05% BSA and containing 15 µg/mL of ApoA1 directly coupled with Alexa Fluor 647 and incubated for 1 h at 4 °C. Stained cells were washed twice in binding buffer and analyzed by flow cytometry.

### MTT assay

A1G and MMG cell lines, mock and GW3965-treated macrophages were seeded in triplicate in 96-well plates at 1 × 10^4^ cells/well for CHO cells and 2 × 10^4^ cells for Raw 264.7 macrophages and incubated in complete Ham’s F-12 medium at 37 °C, 5% CO_2_ overnight to allow the cells to attach to the plate. The medium was then removed, AmB was added in complete Ham’s F-12 medium (or in ΔFBS medium, see next paragraph) in different concentrations (from 0 to 40 µg/mL) and cells were incubated for 3 h at 37 °C. For probucol treatment, the cells were preincubated with 10 µM probucol in complete Ham’s F-12 medium at 37 °C, 5% CO_2_ for 2 h. Then, the medium was removed and AmB was added in different concentrations (from 0 to 40 µg/mL) in complete Ham’s F-12 medium containing 10 µM probucol and cells were incubated for additional 3 h at 37 °C, 5% CO_2_. After incubation, the medium was removed and 3-[4,5-dimethylthiazol-2-yl]-2,5-diphenyltetrazolium bromide (MTT, 0.5 mg/mL, Sigma-Aldrich) in complete Ham’s F-12 was added. Cells were incubated for 4 h at 37 °C, then MTT was removed carefully and the formazan crystals were dissolved in DMSO. Plates were shaken for 10 min before reading them on a UVM 340 microplate reader (Biogenet) at 550 nm with a reference wavelength of 630 nm. The cell viability was estimated as the percentage of the control, which was the cells untreated with any agents (100%).

### Metabolic treatment

To lower the cholesterol content in the cells, A1G and MMG cell lines were seeded at either 1 × 10^4^ cells/well in a 96-well plate (cytotoxicity) or 4 × 10^5^ cells/well (cholesterol content) in a 6-well plate in complete Ham’s F-12 medium for 8 h to allow the cells to attach to the plate. Cells were then washed three times with Ham’s F-12 medium supplemented with delipidated serum (Biowest) (ΔFBS medium) and incubated with 10 µM zaragozic acid (ZA) in ΔFBS medium for 16 h at 37 °C, 5% CO_2_. To reload the plasma membrane with cholesterol, cells after ZA treatment were washed three times with ΔFBS medium and incubated with 100 µM cholesterol–1 mM methyl-β-cyclodextrin complex [31] in ΔFBS medium for 30 min at 37 °C, 5% CO_2_. As a control, cells were incubated for 16 h with only ΔFBS medium at 37 °C, 5% CO_2_. After treatment, cells were either processed with AmB cytotoxicity MTT assay or lysed as described for cholesterol content measurement.

### Cholesterol content measurement

The cells were harvested, washed three times with PBS and lysed with RIPA buffer (25 mM HEPES, pH 7.4, 150 mM NaCl, 1% NP40, 10 mM MgCl_2_, 1 mM EDTA, 2% glycerol, protease and phosphatase inhibitor cocktail) for 30 min on ice. Lysates were then centrifuged at 10,000*g* for 10 min at 4 °C and supernatant was recovered. Total cellular cholesterol level was determined enzymatically by cholesterol oxidase using the Amplex Red Cholesterol Assay Kit (Thermo Fisher Scientific) according to the manufacturer’s recommendations. For cholesterol content estimation, an equivalent of 5 µg of proteins was used. The protein concentration was measured by BCA Assay Kit (Sigma-Aldrich). Samples were mixed with Amplex Red reagent/HRP/cholesterol oxidase/cholesterol esterase working solution and incubated for 30 min at 37 °C under light exclusion conditions. Fluorescence was measured using excitation at 560 nm and emission detection at 590 nm with a Cary Eclipse fluorescence microplate reader (Agilent Technologies). The background was subtracted from the final value. The cholesterol concentration was established using a standard curve. The final cholesterol content was calculated in ng of cholesterol per µg of protein.

### [^3^H]-cholesterol efflux studies

The [^3^H]-cholesterol efflux protocol was adapted from Yang and Gelissen [32]. Briefly, A1G and MMG cells were seeded in 0.5 mL of complete Ham’s F12 medium at 5 × 10^4^ cells/well in 24-well plates and grown overnight at 37 °C, 5% CO_2_. The next day, the cell culture medium was replaced either with 0.25 mL of complete Ham’s F12 medium or complete Ham’s F12 medium containing 1 µCi/mL of [^3^H]-cholesterol (Perkin Elmer) and incubated for 24 h at 37 °C, 5% CO_2_. On the last day, the cell medium was removed, and the cells were washed three times with PBS buffer (with intermediate 15 min incubation in PBS buffer). Afterwards, cells were incubated for 3 h in 0.25 mL of complete Ham’s F12 medium containing (or not, in controls) 20 µg/mL of AmB at 37 °C, 5% CO_2_. For ApoAI efflux, after overnight incubation with [^3^H]-cholesterol (as above), the cells were washed three times with Ham’s F12 medium supplemented with 0.1% of fatty acid-free BSA (Ham’s F12-BSA) (with intermediate 15-min incubation in this medium) and then 0.5 mL of Ham’s F12-BSA containing 10 µg/mL of ApoAI (or not, in controls) was added and incubated 16 h at 37 °C, 5% CO_2_. Finally, the culture medium was recovered, and cells were washed once with 0.1 mL of PBS buffer (pooled with cell medium) and lysed in 0.35 mL of 0.1 M NaOH. One hundred fifty microliters of each of cell medium and cell lysate were added to 5 mL of Ecolite + scintillation cocktail (MPBIO) and radioactivity was counted in CPM mode using a Beckman LS analyzer (Beckman Coulter). After subtraction of background radioactivity, the percentage of [^3^H]-cholesterol efflux was calculated according to the formula: (CPM in media)/[(CPM in media) + (CPM in cells)].

### FLIM analysis

Amphotericin B (AmB) from *Streptomyces* sp. (80% purity) and dimethyl sulfoxide (DMSO) were purchased from Sigma-Aldrich. The procedure of further purification of AmB was described previously in detail [33]. To determine AmB concentration, absorption spectra were recorded with a Cary 60 UV–Vis spectrophotometer (Agilent Technologies). Concentration was established based on the molar extinction coefficient (130,000 M^−1^ cm^−1^) at 408 nm absorption maximum [34].

The cells (A1G and MMG cell lines) were seeded and incubated overnight on sterile 25-mm cover slides (2.5 × 10^5^ cells per slide) in complete Ham’s F12 medium in six-well plates. Before the experiment cells were washed twice with HBSS buffer supplemented with 10 mM HEPES, pH 7.4 (Gibco) and were incubated with 20 µg/mL of AmB in HBSS–HEPES buffer for 30 min at 37 °C and then directly analyzed by FLIM in the presence of AmB.

Fluorescence lifetime imaging was carried out on a MicroTime 200 (Picoquant GmbH, Germany) connected with an Olympus IX71 inverted microscope. The cell samples were illuminated by 405-nm pulsed laser with 10 MHz repetition frequency and 16 ps resolution time. During the experiments, a silicon oil-immersed objective (NA = 1.3, 100 ×) was used. Fluorescence was observed with application of ZT 405RDC dichroic, ZET405 StopLine Notch and 430-nm-long wavelength pass filters from MZ-Analysentechnik. A confocal pinhole of 100 μm in diameter was used. The fluorescence signal was divided into two, perpendicular and parallel polarized channels, and was measured simultaneously by identical single-photon avalanche diodes (τ-SPAD, Picoquant GmbH, Germany). The instrumental correction factor for anisotropy (*G*-factor) was 1.03. Fluorescence lifetime and fluorescence anisotropy values were obtained with the application of SymPhoTime 64 v. 2.3 software (Picoquant GmbH, Germany).

Fluorescence emission spectra of the imaged cells were recorded with the application of a Shamrock 163 spectrograph and a single-photon-sensitive camera (Newton EMCCD DU970P BUF, Andor Technology cooled to minus 50 °C) both coupled to the MT 200 microscope. Application of this system enables detection of fluorescence emission spectra on microscale.

### Mathematical and statistical analysis

All mathematical and statistical analysis was performed using the GraphPad Prism 7 software package (GraphPad). For IC_50_ value determination, a nonlinear fit (inhibitor vs. normalized response with variable slope) of cell viability in the function of AmB decreased concentrations was applied. The statistical significance of differences was assessed either by two-way ANOVA or by *t* test. For all tests, the significance level (alpha, *α*) was set to 0.05. Significant differences are indicated in individual graphs with *p* values as follows: ****p < 0.0001, ***p < 0.001, **p < 0.01, *p < 0.1. If the differences were not significant, nothing is stated. For each result, an appropriate description of the analysis appears in the figure legend.

## Results

### AmB toxicity in Raw 264.7 macrophages and CHO-K1 cell lines stably expressing ABCA1

We first aimed to verify whether the AmB resistance phenomenon occurs in cells expressing ABCA1 endogenously. Macrophages are the principal cell type expressing ABCA1 physiologically as part of the reverse cholesterol transport pathway (RCT) [35]. We used murine Raw 264.7 macrophages that were treated with the LXR agonist GW3965 to induce ABCA1 expression [36, 37]. First, we assessed the ABCA1 expression level after 24-h treatment with 1 µM GW3965 or with mock (DMSO), by flow cytometry using anti-ABCA1 antibody directly coupled to Alexa Fluor 647 and by western blot (Suppl. Fig. 1a, b—upper panel, respectively). Clear induction of ABCA1 expression after agonist treatment was observed. In contrast to ABCA1, the expression of ABCG1 (the closest ABCA1 functional homolog [38]) was stable and not affected by agonist treatment (Suppl. Fig. 1b, middle panel).

**Fig. 1:**
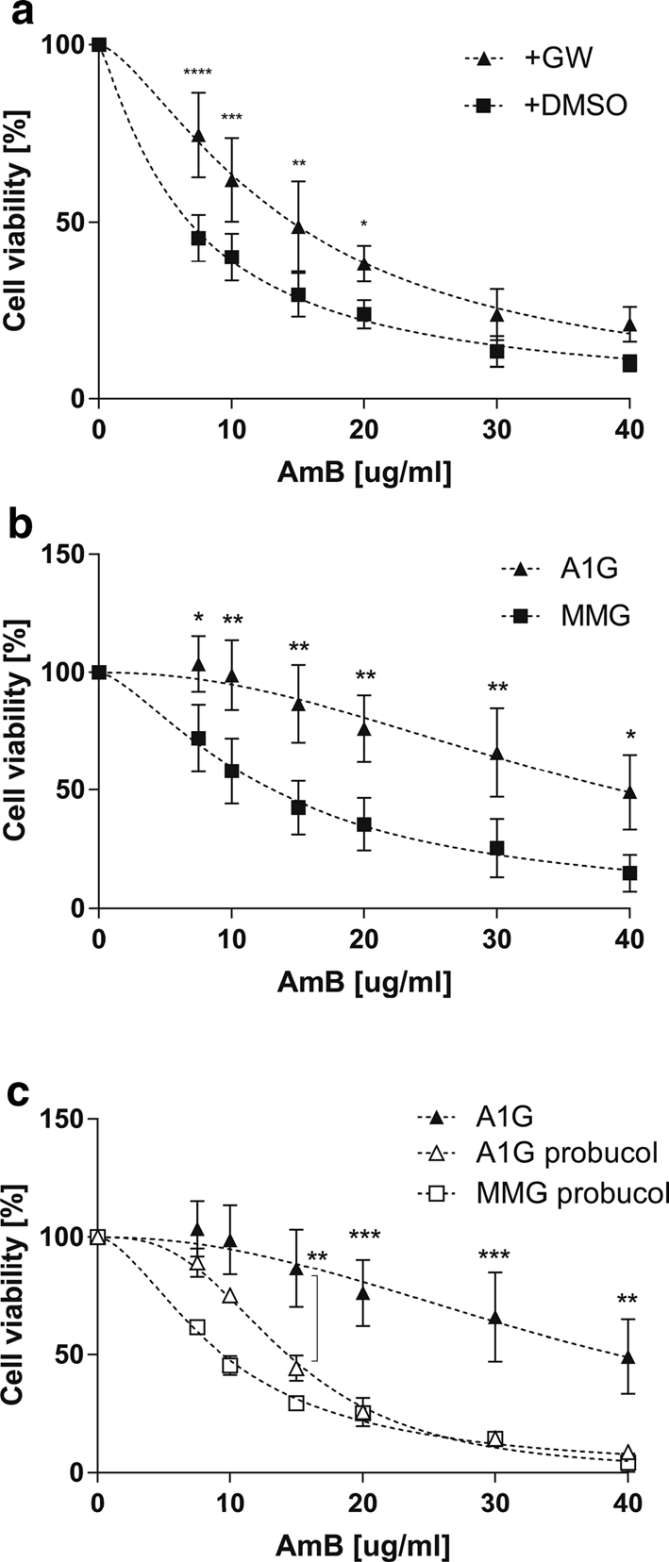
AmB cytotoxicity in Raw 264.7 macrophages and CHO-K1 cell lines stably expressing ABCA1. **a** Graph representing a nonlinear fit (dashed line) of cell viability as a function of decreased AmB concentrations assessed by MTT assay in Raw 264.7 cells treated or not with GW3965. Axes represent AmB concentration in µg/mL (*x* axis) and cell viability in percent of control (*y* axis). Two-way ANOVA (*α* 0.05) with Sidak’s multiple comparison test was used to assess the significance of differences (****p < 0.0001, ***p < 0.001, **p < 0.01, *p < 0.1). Error bars correspond to SD. The data were obtained in four independent experiments, each in triplicates. **b** Graph representing a nonlinear fit (dashed line) of cell viability as a function of decreased AmB concentrations assessed by MTT assay in A1G and MMG cells. Axes represent AmB concentration in µg/mL (*x* axis) and cell viability in percent of control (*y* axis). **c** Graph representing a nonlinear fit (dashed line) of cell viability as a function of decreased AmB concentrations assessed by MTT assay in A1G and MMG cells treated with 10 µM probucol. The data of non-probucol-treated A1G cells come from **b**. Axes represent AmB concentration in µg/mL (*x* axis) and cell viability in percent of control (*y* axis). Two-way ANOVA (*α* 0.05) with Sidak’s multiple comparison test was used to assess the significance of differences (***p < 0.001 **p < 0.01, *p < 0.1). Error bars correspond to SD. The data were obtained in three (**b**) or two (**c**) independent experiments, each in triplicates

Amphotericin B cytotoxicity towards Raw 264.7 macrophages (treated with GW3965 or mock control) was tested and cell viability was revealed using MTT (3-(4,5-dimethylthiazol-2-yl)-2,5-diphenyltetrazolium bromide) assay. Cells were exposed to increased concentrations of AmB, ranging from 0 to 40 µg/mL, for 3 h and then the viability was analyzed by MTT assay. As shown in Fig. 1a, mock control Raw 264.7 cells were more sensitive to AmB treatment then the cells incubated with GW3965 prior to the experiment. A significant effect of mock cells’ sensibility compared to ABCA1-expressing cells was observed, starting from a low concentration of AmB. The calculated IC_50_ values were 6.18 µg/mL (SE ± 0.46) for control and 14.55 µg/mL (SE ± 0.78) for agonist-induced Raw 264.7 macrophages.

To further investigate observations obtained in Raw 264.7 macrophages and confirm that the AmB resistance is directly linked to the expression of ABCA1, we generated CHO-K1 clonal cell lines stably expressing either an active ABCA1-GFP (A1G) protein or a non-active ABCA1MM-GFP (MMG) catalytic mutant [23] whose expression was driven under the control of a moderate phosphoglycerate kinase (*Pgk*) promoter [29]. The expression levels of A1G and MMG stable lines assessed by FACS and western blot are shown in Supplementary Fig. 2a, b. Both proteins were expressed, but the MMG expression level was higher than that of A1G. The lower expression level of A1G might be explained by increased toxicity of ABCA1 protein when stably expressed. It is notable that there was also very low but detectable expression of endogenous ABCA1 in wild-type CHO-K1 cells. We also analyzed the endogenous expression of ABCG1, which similarly to Raw 264.7 was comparable between CHO-K1 cells and ABCA1-expressing lines (Suppl. Fig. 2b, middle panel). Confocal analysis of A1G and MMG cell lines showed that ABCA1 protein is correctly localized to the PM with a fraction of protein localized in intracellular membranes (Suppl. Fig. 2c). To assess the functionality of ABCA1 in stable lines, we performed an apolipoprotein AI (ApoAI)-binding assay and efflux of radiolabeled [^3^H]-cholesterol to ApoAI. ApoAI is a natural acceptor of cholesterol and phospholipids in an ABCA1-dependent manner in the reverse cholesterol pathway and binds to cells expressing active ABCA1 [16, 17]. In Supplementary Fig. 2d, we can see that only A1G-expressing cells strongly bind ApoAI (ApoAI–AF647), in contrast to MMG cells, confirming that ABCA1 protein in the A1G cell lines is fully functional and the GFP tag does not impair its function. Moreover, the lack of ApoAI binding by MMG cells suggests that low endogenous expression of ABCA1 coming from the CHO-K1 background is negatively dominated by MMG phenotype [39]. In addition, A1G cells were able to promote efflux of radiolabeled [^3^H]-cholesterol to ApoAI in contrary to MMG cells (Suppl. Fig. 2e). The A1G and MMG cell lines were then exposed to increased concentrations of AmB ranging from 0 to 40 µg/mL for 3 h at 37 °C, and then cell viability was analyzed by MTT assay. As shown in Fig. 1b, MMG cells were much more sensitive to AmB treatment than A1G cells. The difference in sensitivity was significant starting from a low AmB concentration. The calculated IC_50_ values were 39.2 µg/mL for A1G and 13.1 µg/mL for MMG (Table 1), showing that IC_50_ for A1G is three times higher than in the case of MMG cells. These results were concordant with our previous observation in Raw 264.7 macrophages. In addition, CHO-K1 wild-type cells displayed similar sensitivity towards AmB as MMG cells, with an IC_50_ value of 16.75 µg/mL (SE ± 1.21) (Suppl. Fig. 3). Finally, we investigated the influence of ABCA1 inhibitor probucol on cell sensitivity towards AmB. Probucol has been reported as a specific inhibitor of ABCA1 and ABCA1-mediated cholesterol efflux from cells without any impact on ABCA1 protein level [40–42]. The A1G and MMG cells were pretreated during 2 h with 10 µM probucol and then were exposed to increased concentrations of AmB ranging from 0 to 40 µg/mL for 3 h at 37 °C in the presence of 10 µM probucol. The cell viability was analyzed by MTT assay. As it is shown in Fig. 1c, probucol-treated A1G cells showed a significant increase in sensitivity to AmB starting from a concentration of 15 µg/mL when compared to non-treated A1G cells. The calculated IC_50_ values in respect to AmB and after probucol treatment were 14.36 µg/mL for A1G and 9.47 µg/mL for MMG cells, respectively (Table 1).

**Table 1:** Summary of IC_50_ values for AmB in A1G and MMG cells

IC_50_ (µg/mL)	A1G	MMG
Complete Ham’s F12	**39.2** ± 4.49	**13.1** ± 1.0
6 h AmB	**30.93 **± 1.66	**10.59 **± 0.48
16 h AmB	**11.44 **± 0.35	**6.65 **± 0.28
+ 10 µM probucol	**14.36 **± 0.36	**9.47 **± 0.31
ΔFBS medium	**21.18** ± 1.09	**10.15** ± 0.42
ΔFBS medium + ZA	**9.75** ± 0.53	**10.04** ± 0.67
ΔFBS medium + ZA + Chol.	**18.88** ± 0.77	**6.42** ± 0.38

Table represents a comparison of IC_50_ values for AmB expressed between A1G and MMG cells calculated from a nonlinear fit of cell viability as a function of decreased AmB concentrations in cytotoxicity experiments performed in complete Ham’s F12 medium during 3 h (complete Ham’s F12), complete Ham’s F12 medium during 6 h (6 h AmB), complete Ham’s F12 medium during 16 h (16 h AmB), complete Ham’s F12 medium with 10 µM probucol (+ 10 µM probucol), ΔFBS medium, ΔFBS medium with zaragozic acid (ΔFBS medium + ZA), and ΔFBS medium with ZA, and reloaded with cholesterol (ΔFBS medium + ZA + Chol). The IC_50_ values (in bold) are expressed in µg/mL ± SE

### FLIM imaging reveals specific AmB structures in A1G cells

**Fig. 2:**
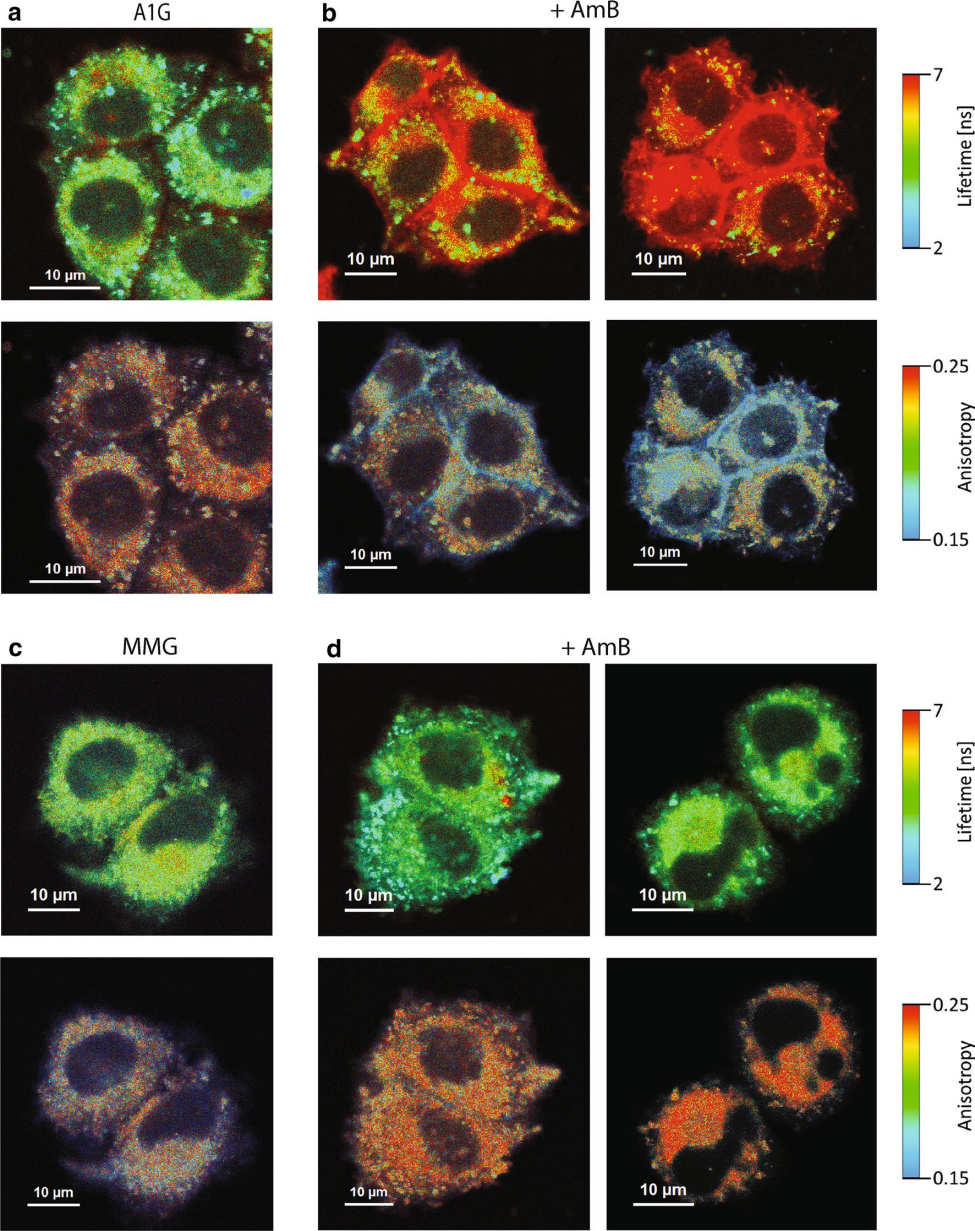
FLIM imaging of AmB molecular organization in ABCA1-expressing cell lines. **a** A1G cells’ fluorescence lifetime and fluorescence anisotropy images (upper and bottom panels, respectively). **b** Fluorescence lifetime and fluorescence anisotropy images of A1G cells (two examples) treated for 30–60 min with 20 µg/mL of AmB (upper and bottom panels, respectively). **c** MMG cells’ fluorescence lifetime and fluorescence anisotropy images (upper and bottom panels, respectively). **d** Fluorescence lifetime and fluorescence anisotropy images of MMG cells (two examples) treated for 30–60 min with 20 µg/mL of AmB (upper and bottom panels, respectively). Fluorescence lifetime (ns) and anisotropy scales are represented at the right of each image panel. Scale bars correspond to 10 µm

Next, to better understand the phenomenon of ABCA1-mediated resistance to AmB, we decided to investigate the AmB penetration into cells and molecular organization of the antibiotic molecules with application of fluorescence lifetime imaging microscopy (FLIM). FLIM analysis was performed on A1G and MMG cells, and the same cell lines exposed to interaction with AmB (Fig. 2a–d, upper panels). Excitation of the samples with blue light laser (405 nm) gives rise to a relatively high fluorescence signal. A large fraction of this signal originates from light emission by GFP and the cell autofluorescence, as can be seen from the images of control cells, presented in Fig. 2a, c (upper panels). Most of this emission is characterized by the fluorescence lifetime ~ 3 ns and is represented by green color in the images. As can be seen from the cell images, the presence of AmB (20 µg/mL for 30–60 min) in the A1G cells is associated with pronounced effects both in the images representing fluorescence lifetime (Fig. 2b, upper panels) and in those representing anisotropy (Fig. 2b, bottom panels). A relatively intense fluorescence signal in the cell surface region, characterized by a long fluorescence lifetime (> 6 ns), coded with red color (Fig. 2b, upper panels), can be assigned to antiparallel dimeric structures of AmB [43, 44]. The same structures are characterized by relatively low fluorescence anisotropy values (see the bottom panels in Fig. 2b), which is consistent with high motional freedom of the molecular structures of AmB formed in the surface zone of the cell membranes. The fact that such structures are not formed in the MMG cells lacking the ABCA1 catalytic activity (Fig. 2d), able to liberate cholesterol molecules from the cell membranes and expose them to interaction with AmB, suggests that molecular structures of the drug manifested in the FLIM images presented in Fig. 2b (A1G cells) are preferentially formed in the environment of cholesterol and possibly contain the sterol as an element of such structures. It has been demonstrated that presence of either ergosterol or cholesterol in the lipid membranes was necessary for incorporation of AmB into the hydrophobic core of the membranes [43]. In the case of the MMG cells exposed to AmB, an increase of the fluorescence anisotropy can be observed (Fig. 2d, bottom panels), which is diagnostic for immobilization of AmB molecular structures and corroborates incorporation of the drug into the membranes.

**Fig. 3:**
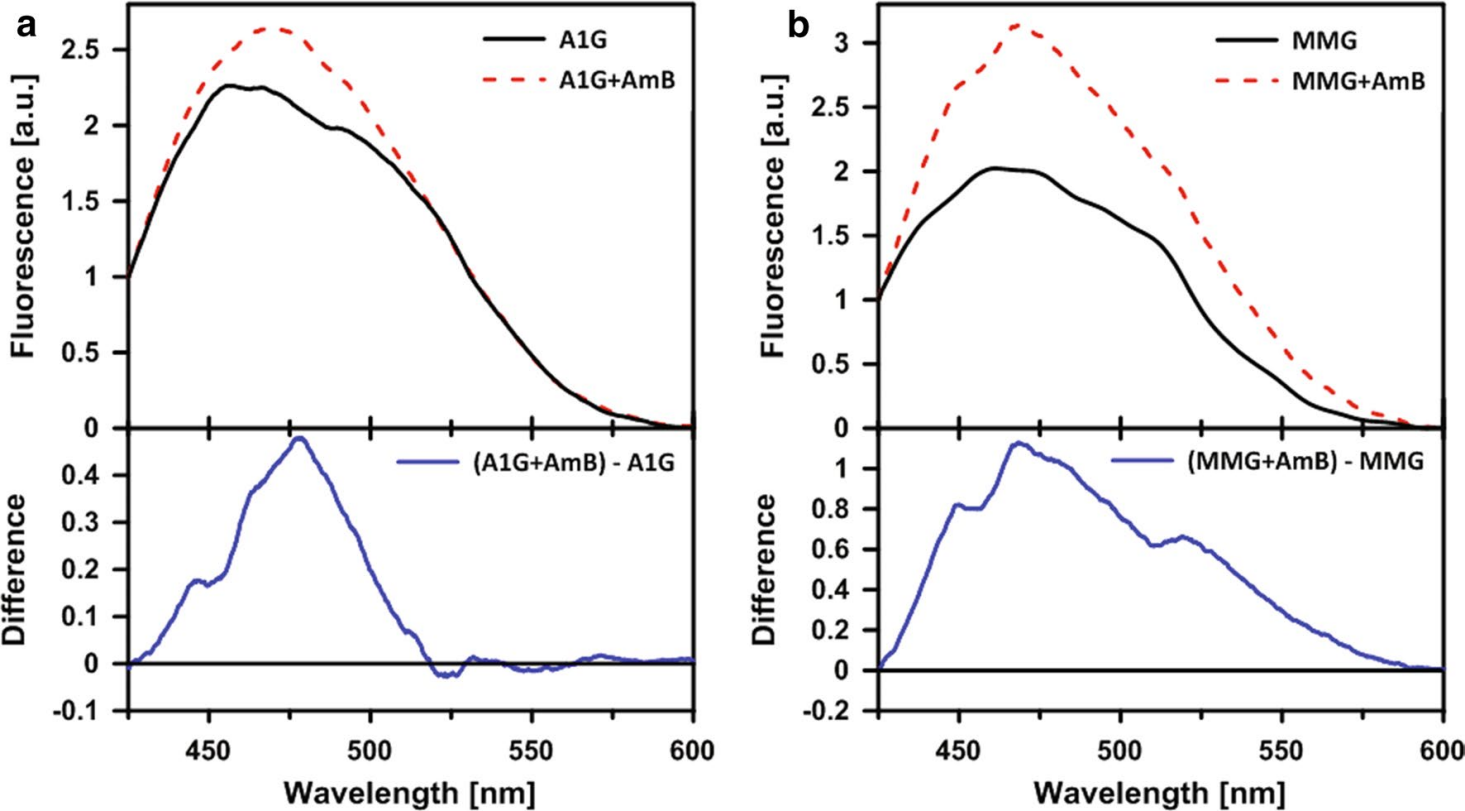
Micro-spectroscopy measurements of fluorescence emission spectra recorded at the surface of A1G cells and MMG cells. Fluorescence emission spectra recorded at the surface of A1G cells (**a**) and MMG cells (**b**). Upper panels represent fluorescence emission spectra of cells without AmB (black line) and after addition of AmB (red dashed line). The original fluorescence emission spectra were normalized at 425 nm. Bottom panels represent fluorescence emission spectra resulting from subtraction of fluorescence spectra without AmB from fluorescence spectra after AmB addition

Analysis of the fluorescence emission spectra recorded at the surface of cells (Fig. 3a, b, upper panels) shows that AmB binds to cells representing both the cell lines, A1G and MMG, but molecular organization of the drug is different. This can be directly seen from comparison of the difference in spectra representing AmB spectral forms in the A1G and MMG cells (Fig. 3a, b, bottom panels). In both cases, fluorescence of AmB shows a relatively broad band between 430 and 500 nm. This band can be assigned as representing molecular structures of the antibiotic, with light emission shifted towards longer wavelengths, owing to excitonic interactions, in particular dimers, both parallel and antiparallel [44]. The difference spectrum representing AmB bound to MMG cells has an additional, long-wavelength band, in the spectral region between 500 and 600 nm (Fig. 3b, bottom panel). This particular band can be assigned to higher dimension molecular structures of AmB, including tetramers, represented by short fluorescence lifetimes and able to penetrate sterol-rich lipid membranes [43]. Such structures are considered as responsible primarily for disturbance of the ionic equilibrium of cell membranes.

The fact that AmB forms supramolecular bulk structures outside the cells, in the case of A1G cell lines with active cholesterol transport out of the cells, can help to understand relatively high cell viability when exposed to AmB compared to the MMG cell lines (Fig. 1b). In the latter case, cholesterol remains in the hydrophobic core of the cellular membrane, providing favorable conditions for internalization of the drug into the membrane, which implies toxicity of AmB and a related decrease in viability.

### A1G promotes efflux of [^3^H]-cholesterol to AmB

**Fig. 4:**
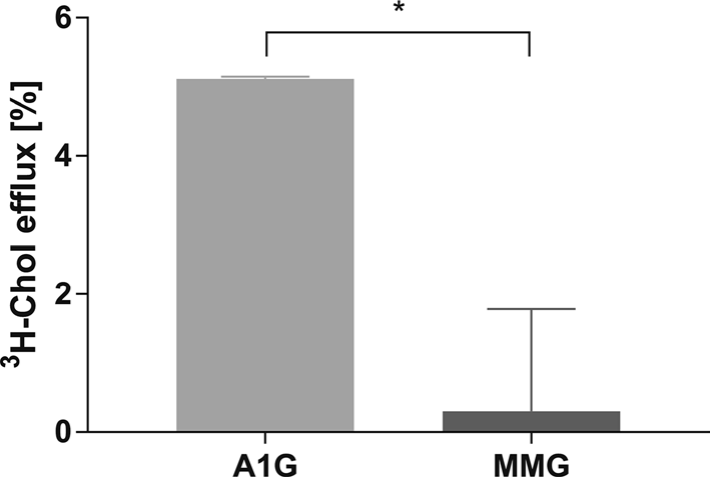
A1G-mediated [^3^H]-cholesterol efflux to AmB. Graph representing [^3^H]-cholesterol efflux to AmB as an acceptor in A1G and MMG cells expressed in percent of efflux (*y* axis). The significance of difference in efflux between A1G vs. MMG was validated with two-tailed *t* test at the confidence level of 95% (*p < 0.05). Error bars correspond to SD. The data were obtained in two independent experiments, each in duplicates

We wanted to extend our FLIM observations and verify whether AmB may play the role of a cholesterol acceptor promoting its efflux from A1G cells. We performed [^3^H]-cholesterol efflux studies using AmB as an artificial acceptor. The cells were loaded with radiolabeled cholesterol for 24 h and then efflux to AmB was performed for 3 h. As shown in Fig. 4, A1G cells show significantly higher cholesterol efflux to AmB (5.1%) compared to MMG cells (0.3%). This observation suggests that indeed, AmB may help in the liberation of cholesterol molecules from A1G-expressing cell membranes and then interact with them to form cholesterol–AmB aggregates from which AmB is unable to cross the plasma membrane and cause cellular toxicity.

### AmB resistance phenomenon is abolished by partial cholesterol depletion in cells

**Fig. 5:**
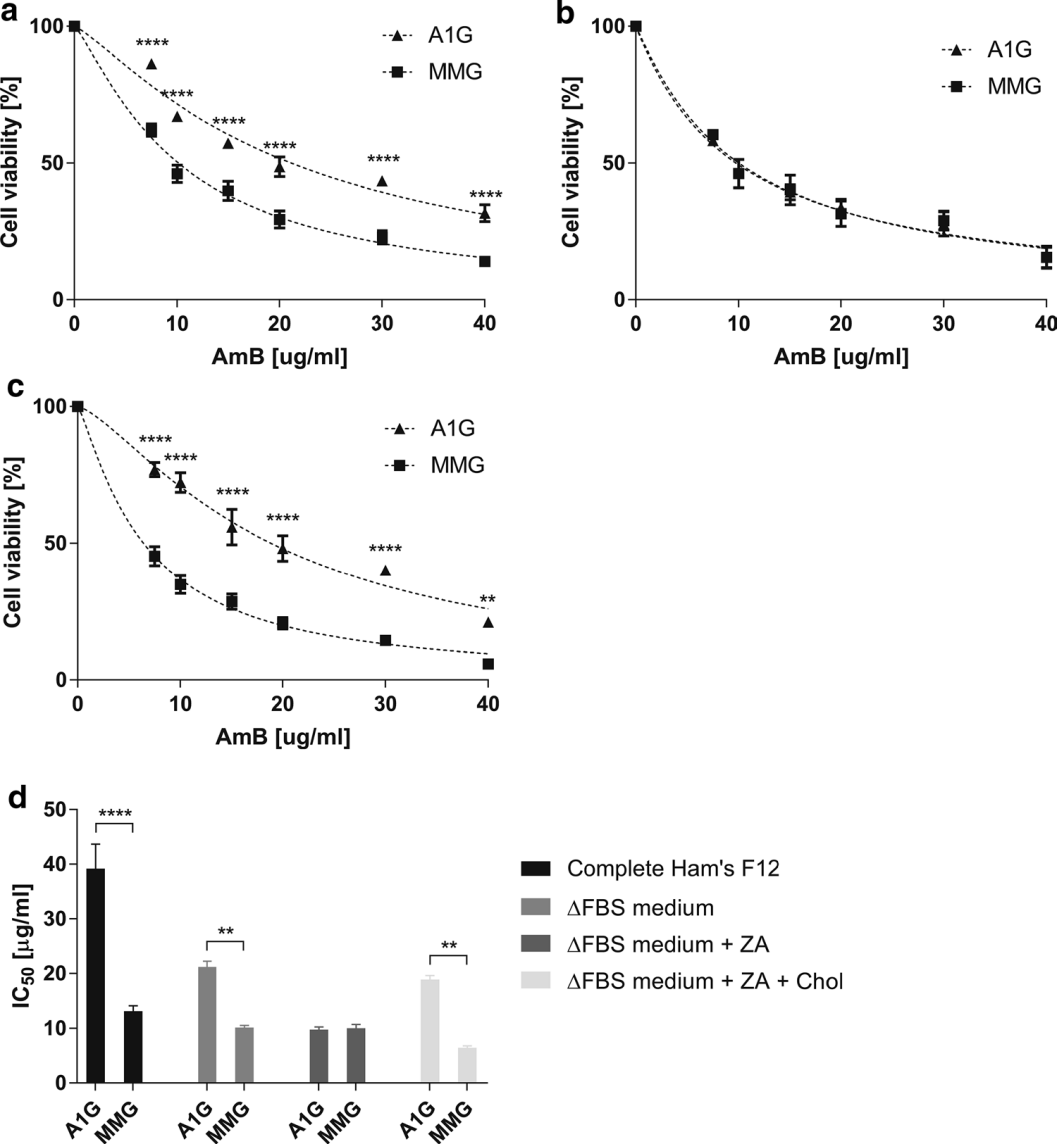
Influence of cholesterol content on ABCA1-dependent resistance to AmB. Graphs represent a nonlinear fit (dashed line) of cell viability as a function of decreased AmB concentrations assessed by MTT assay in A1G and MMG cells cultured in ΔFBS medium (**a**), ΔFBS medium with zaragozic acid (ZA) (**b**), ΔFBS medium with ZA and reloaded with cholesterol (**c**). Axes represent AmB concentration in µg/mL (*x* axis) and cell viability in percent of control (*y* axis). Two-way ANOVA (*α* 0.05) with Sidak’s multiple comparison test was used to assess the significance of differences (****p < 0.0001, **p < 0.01). Error bars correspond to SD. The data were obtained in two independent experiments, each in triplicates. **d** Graph representing a comparison of IC_50_ values for AmB between A1G and MMG cells calculated from a nonlinear fit of cell viability as a function of decreased AmB concentrations in cytotoxicity experiments performed in complete Ham’s F12 medium, ΔFBS medium, ΔFBS medium with zaragozic acid (ΔFBS medium + ZA), ΔFBS medium with ZA and reloaded with cholesterol (ΔFBS medium + ZA + Chol) presented in Fig. 1b and this figure **a**–**c**. Two-way ANOVA (*α* 0.05) with Sidak’s multiple comparison test was used to assess the significance of differences (****p < 0.0001, **p < 0.01). Error bars correspond to SEM

Since cholesterol is a major substrate of ABCA1, we aimed to determine how the cellular cholesterol level may modulate the ABCA1-induced resistance to AmB. For this purpose, we performed cytotoxicity tests on cells that were cultivated either in delipidated serum (ΔFBS medium) or in which cholesterol synthesis was metabolically blocked by zaragozic acid (ZA) treatment. Zaragozic acid is a potent inhibitor of squalene synthase [45], an enzyme catalyzing condensation of two farnesyl pyrophosphate molecules into a squalene molecule, one of the major steps in the cholesterol biosynthesis pathway. ZA treatment of cells results in a significant decrease of total cholesterol content, including the plasma membrane cholesterol fraction [46]. The cells cultivated in ΔFBS medium showed a significant decrease in cholesterol content (Suppl. Fig. 5). However, in these conditions, A1G cells still displayed considerable resistance toward AmB compared to MMG cells (Fig. 5a; Table 1). The cell treatment with ZA (in ΔFBS medium) completely abolished the differences between these two lines in terms of AmB sensitivity (Fig. 5b; Table 1). However, reload of ZA-treated cells with methyl-β-cyclodextrin-complexed cholesterol restored the resistance phenomenon of A1G cells (Fig. 5c; Table 1) to a level comparable with that in ΔFBS medium. It is important to underline that the resistance of A1G cells, but not MMG cells, correlates with the cholesterol content in cells (Fig. 5d; Suppl. Fig. 5). In standard culture conditions, the IC_50_ for A1G was 39.2 µg/mL, then it decreased to a value of 21.18 µg/mL in ΔFBS medium and finally dropped to 9.75 µg/mL after ZA treatment. The cholesterol reload raised it back to a value of 18.88 µg/mL (Fig. 5d; Table 1; Suppl. Fig. 5). Pearson’s correlation coefficient *r* calculated from these data is 0.9 in the case of A1G cells, which indicates a strong correlation between cellular cholesterol content and AmB cytotoxicity, whereas for MMG cells it is minus  0.22, indicating no correlation.

Another interesting observation was that after reload with cholesterol, MMG cells were even more sensitive to AmB treatment when compared to cells cultivated in ΔFBS medium (Suppl. Fig. 6). A possible explanation of this observation would be that since the MMG cells are not able to efflux cholesterol loaded at the PM, its local accumulation would be then a direct site of increased AmB binding and provoking cellular toxicity.

## Discussion

Despite the huge progress of investigations in the field of AmB interactions with membranes, only a small fraction of them have concentrated on AmB–plasma membrane interactions in living cells. Since AmB is still the main anti-mycotic agent used in the treatment of immunosuppressed and transplanted patients [47–49], it is extremely important to understand the molecular mechanism leading to its cytotoxicity towards mammalian cells. Here, we propose a new potential mechanism of cell resistance to AmB based on the activity of the cholesterol efflux protein ABCA1.

ABCA1 is a lipid transporter governing a reverse cholesterol pathway in mammals by promoting cholesterol removal from peripheral tissues to the liver. This involves mobilization of intracellular cholesterol making it available for plasma acceptors such as ApoAI at the outer leaflet of the PM [16, 17, 50]. Several lines of evidence suggest that this involves displacement of cholesterol pools within the PM. Indeed, it has already been shown that ABCA1 actively redistributes cholesterol from lipid-raft nanodomains into smaller pools, and this is crucial for ApoAI binding and generation of HDL molecules [25, 26, 51–53]. ABCA1 would then create a particular nano-environment at the PM where plasmatic receptors such as ApoAI may bind and be subsequently lipidated with phospholipids and cholesterol. Recent structural data suggest that phospholipids are directly transported by ABCA1 flopping from the inner to the outer leaflet of the PM and then to ApoAI bound nearby. Cholesterol efflux to ApoAI would be a concomitant process being a result of phospholipid transport [22]. In this context, a potential role of ABCA1 in the cell membrane interactions with AmB seemed very plausible.

Our initial observation was that increased resistance of Raw 264.7 macrophages and CHO-K1 cells to AmB treatment is strongly correlated with ABCA1 expression (Fig. 1a, b). We believe that this phenomenon is specific to ABCA1 for several reasons. First, the cells without ABCA1 expression or expressing the non-functional ABCA1MM mutant were much more sensitive to AmB treatment. Second, ABCG1, which is the closest ABCA1 functional homolog, also involved in cholesterol efflux [54], had the same expression level in all tested cell lines (Suppl. Figs. 1b and 2b, middle panels) and, therefore, would not differentially influence the AmB cellular resistance. We cannot exclude the possibility that drug efflux pumps participate in AmB removal [55]. However, the majority of drug efflux pump substrates in mammals are compounds removed from cell cytoplasm [56]. This is not the case of AmB, which remains in the core of the PM. Moreover, here the cells were treated with AmB for max. 16 h, which is a very short time and probably not sufficient to develop multidrug resistance [57]. Finally, if it were the case, it should be apparent to the same extent in both A1G and MMG cells. The last evidence that the AmB resistance is related directly to ABCA1 was the observation made on A1G cells treated with ABCA1 inhibitor, probucol. We observed that in these cells the resistance phenomenon is abolished with a significant decrease of A1G cells IC_50_ value in respect to AmB (Fig. 1c; Table 1).

An important element in the exploration of A1G-mediated AmB resistance was given by the studies of AmB molecular organization using FLIM and micro-spectroscopy (Figs. 2, 3, respectively). According to the current knowledge and our understanding, there are two main modes of action of AmB with respect to biomembranes: the first one, which can be referred to as “intramembranous” and the second one as “extramembranous”. In both modes sterol molecules seem to play a critical role. The intramembranous mechanism is associated with formation of transmembrane channels affecting physiological ion transport. Although AmB can form ion channels even in the membranes lacking sterols, as clearly demonstrated by Huang et al. [58], cholesterol and particularly ergosterol promote the incorporation of AmB into lipid bilayers perpendicular with respect to the plane of the membrane [43]. The extramembranous mechanism can operate independently of the intramembranous mode of incorporation. This mechanism is based on the extraction of sterol molecules from biomembranes and immobilizing them in extramembranous AmB–sterol supramolecular structures, referred to as “sponges” [7, 59].

Application of the FLIM technique in the present study combined with fluorescence micro-spectroscopy enabled us to analyze in the same time the localization of AmB in cells exposed to the antibiotic and decipher selectively the mode of molecular organization of the antibiotic. The results show that on the one hand AmB binds to the membranes but on the other hand is also involved in formation of extramembranous structures (Figs. 2, 3, respectively). Interestingly, formation of extramembranous structures of AmB has been selectively observed upon expression of a functional ABCA1 membrane transporter in the presence of AmB. This suggests that this process is not associated with a passive extraction of cholesterol molecules from the lipid bilayer but rather can be described as the formation of two-component sterol–AmB structures from cholesterol molecules actively tackled out of the membranes by a functional ABCA1. These structures, composed mainly of AmB antiparallel dimers, are characterized by a relatively high fluorescence lifetime and lower fluorescence anisotropy, and are found exclusively at the periphery of A1G cells (Fig. 2b) [34, 43]. According to our results, we favor that AmB dimers dominate among the molecular organization form but unfortunately we are currently not able to conclude on a stoichiometry of AmB and cholesterol in such extramembranous structures. Interestingly, molecular organization forms of AmB composed of greater number of molecules, characterized by shorter fluorescence lifetimes, can be identified within the membranes of MMG cells (Fig. 2d), thus indicating possible ion-forming activity of AmB [58]. Therefore, this ABCA1-mediated mechanism can be considered as protective against AmB cell toxicity. Recent study indicated a different defense mechanism, in which human colon cell lines minimize toxic effects of AmB by extrusion of the drug molecules from their membranes via formation of AmB-rich exosomes [60]. However, the participation of any protein in this mechanism is not documented so far.

The cytotoxicity of the AmB–sterol “sponges” depends on the relative AmB-to-sterol proportion, namely when the AmB-to-sterol molar ratio is greater than one [7, 59, 61]. Therefore, the strength of AmB cytotoxicity may depend not only on ABCA1 and its membrane cholesterol transport but also as a function of time and how the two mechanisms (intramembranous and extramembranous) coexist simultaneously.

We indeed showed that at high concentrations of AmB, the amount of cholesterol fluxed by ABCA1 is not sufficient for complexing all AmB molecules out of the PM. This may explain the lower or even the lack of significance in cell viability at high concentrations of AmB in the two cell types (Fig. 1a, b). On the other hand, the increase of AmB time exposure also influences A1G cell viability (Fig. 1b; Suppl. Fig. 4; Table 1). While 6 h of AmB treatment decreases slightly A1G cell viability when compared to 3-h treatment (Fig. 1b; Suppl. Fig. 4a; Table 1), a longer time exposure (16 h) resulted in a strong increase of A1G cell sensitivity to AmB (Suppl. Fig. 4b; Table 1). In our view, a long time exposure to AmB will first cause a decrease of membrane cholesterol content due to the ABCA1-mediated cholesterol efflux to AmB outside of the PM. Therefore, the low cholesterol membrane content limits AmB insertion into the core of the PM for channel formation and promotes the sterol “sponge” extraction mechanism of the drug. At some point, long-term membrane cholesterol lowering will affect the organization and integrality of the PM leading to cell death.

Our last finding was that the AmB resistance phenomenon of A1G cells depends strictly on the cholesterol content in the cell. This observation is a direct confirmation of results discussed above. There was no significant difference of basal cholesterol level between A1G and MMG cells in all tested conditions (Suppl. Fig. 5). The A1G cells cultured in ΔFBS medium had a partial decrease of total cholesterol content with a significant impact on AmB IC_50_ value, which decreased (Fig. 5a; Suppl. Fig. 5; Table 1). However, A1G cells still displayed the AmB resistance phenomenon. Additional treatment with zaragozic acid resulted in approximately 50% decrease of total cellular cholesterol content when compared with non-ZA-treated cells cultured in ΔFBS medium (Suppl. Fig. 5). This decrease resulted in complete abolishment of AmB resistance of A1G cells with the same sensitivity towards AmB as MMG cells (Fig. 5b; Table 1). The AmB resistance of A1G cells was, however, rescued after cholesterol reload (Fig. 5c; Suppl. Fig. 5; Table 1). Taking into consideration that cholesterol is a major substrate of ABCA1 [62], we can assume that at the low cellular cholesterol content, the ability of A1G cells to efflux a sufficient amount of cholesterol to form protective bulk cholesterol–AmB structures would be strongly affected. However, the low cholesterol level would still sustain AmB cytotoxicity mainly by a sterol “sponge” extraction mechanism. This also proves that the ABCA1-mediated cholesterol efflux is a principal reason for the AmB resistance phenomenon, since in MMG cells unable to efflux a cholesterol reload resulted in an additional significant increase of cell sensitivity to AmB (Suppl. Fig. 6).

It has already been reported that ABCA1 expressed in yeast *S. cerevisiae* slightly increases sensitivity of cells towards AmB [63], which is opposite to our recent findings. The molecular mechanism of this observation, however, has not been investigated in detail, and it was not confirmed that ergosterol is indeed transported by ABCA1 in yeast. Moreover, the yeast cells were grown in the presence of AmB for more than 20 h and the sensitivity of yeast expressing active ABCA1 started to appear after 6 h of culture. The long exposure of yeast to AmB upregulates expression of several gene classes involved in cell detoxification including ABC transporters [64]. It is possible that some of them may interfere with ABCA1 activity. The main structural difference of yeast PM is that it contains ergosterol and has different spatial organization compared to mammals [65]. It is well established that AmB interacts more efficiently with ergosterol-containing membranes binding more rapidly and forming larger and more stable oligomeric structures inside the membrane (with usually ten times lower IC_50_ compared to cholesterol-containing membranes) [6, 34, 66]. In this view, we can assume that the expression of ABCA1 in yeast cells may have a different outcome when compared with mammalian cells and cannot be directly related to our observations. It is possible that active ABCA1 expressed in yeast will somehow modify plasma membrane organization, possibly without ergosterol efflux, and this would imply an increase in sensitivity to AmB. It has also been reported that overexpression in the yeast *S. cerevisiae* of CDR1 and CDR2 ABC transporters from *C.* *albicans* resulted in increased sensitivity to AmB [67].

**Fig. 6:**
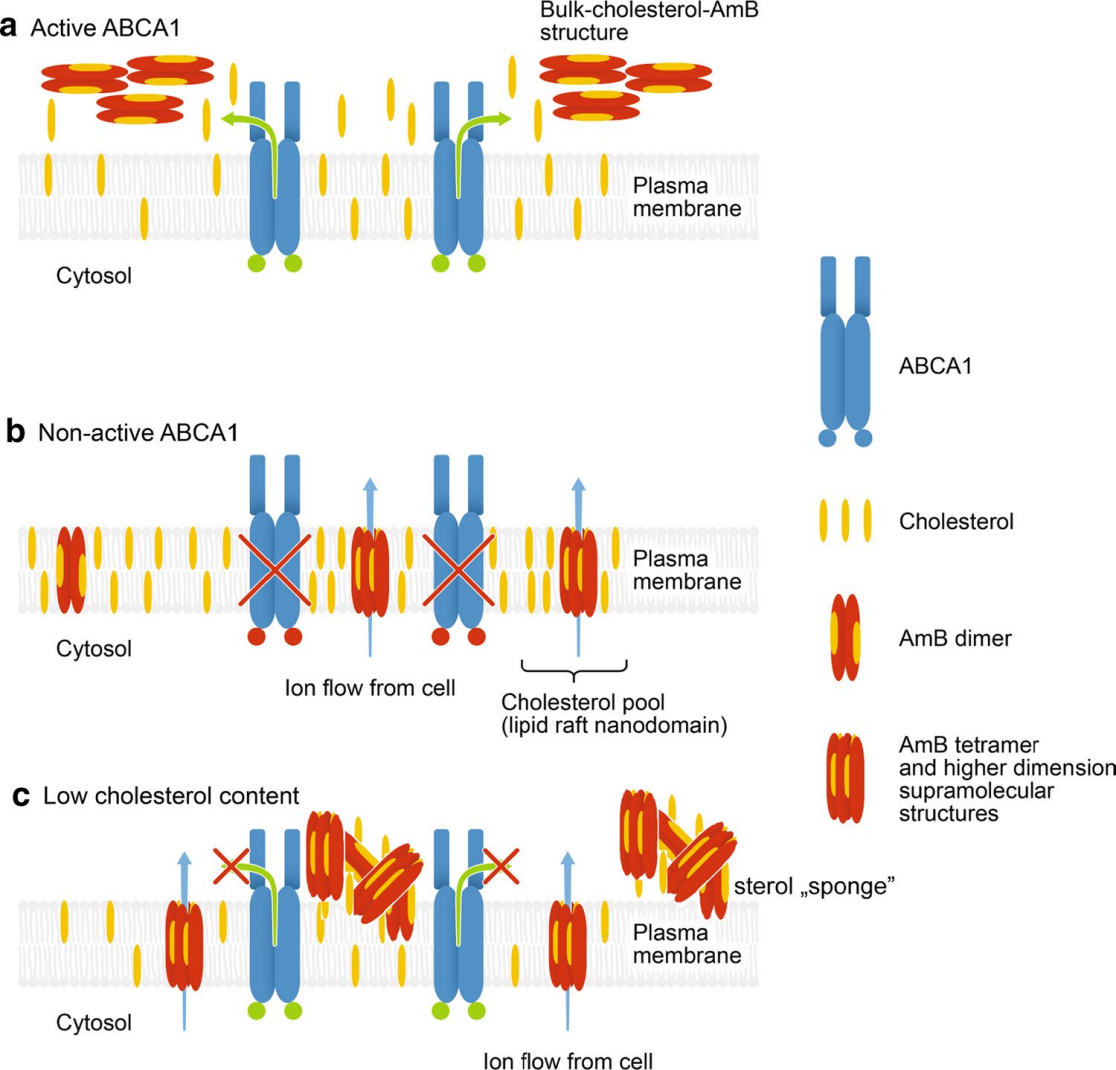
Model of ABCA1-dependent cell resistance to AmB. **a** When ABCA1 is active (green dots), it mediates the efflux of cholesterol from plasma membrane to AmB. This leads to dimerization of AmB at the cell surface and formation of bulk cholesterol–AmB structures from which AmB is not able to penetrate the plasma membrane and cause cellular cytotoxicity. **b** Inactive ABCA1 (red dots) or lack of ABCA1 protein (red cross) will not change the PM cholesterol content and organization. In these conditions, AmB will incorporate into the lipid bilayer of plasma membrane, forming active dimers and tetramers, and resulting in cellular toxicity. **c** Low cholesterol level in the cell will affect to some extent the efflux activity of ABCA1, which will result in a lack of protective bulk cholesterol–AmB structure formation, and will lead to AmB cytotoxicity mainly by sterol “sponge” mechanism

To summarize, we propose a simplified model of ABCA1-mediated resistance to AmB. When ABCA1 is present and active at the plasma membrane (Fig. 6a), it facilitates efflux of membrane cholesterol to AmB, leading to the formation of bulk cholesterol–AmB structures and, therefore, prevents AmB cytotoxicity. When there is no ABCA1 or it is inactive (Fig. 6b), cholesterol remains in the core of the plasma membrane, usually localized in cholesterol-rich raft nanodomains, where AmB can insert and form active oligomeric structures promoting cellular cytotoxicity. Finally, a low cholesterol level in the cell will affect to some extent the efflux activity of ABCA1, which will result in a lack of formation of protective bulk cholesterol–AmB structures at the cell surface and will lead to AmB cytotoxicity mainly by sterol “sponge” mechanism (Fig. 6c).

A final but important consideration is potential application of our findings in the future, i.e. in AmB treatment of patients after transplantation. We can envisage that ABCA1 expression might be pharmacologically upregulated, by, for example, LXR pathway agonists or statins [68, 69], which may result in increased resistance of patient cells to AmB and decreased cytotoxic effects of the drug during treatment.

### Electronic supplementary material

Below is the link to the electronic supplementary material.

Supplementary material 1 (DOCX 4667 kb)

## Abbreviations

A1G: ABCA1-GFP
ABC: ATP-binding cassette transporter
AmB: Amphotericin B
ApoAI: Apolipoprotein AI
CHO-K1: Chinese hamster ovary K1 cells
eGFP: Enhanced green fluorescent protein
FACS: Fluorescence-activated cell sorting or flow cytometry
FLIM: Fluorescence lifetime imaging microscopy
HDL: High-density lipoprotein
LXR: Liver X receptor
MMG: ABCA1MM-GFP
MTT: 3-[4,5-Dimethylthiazol-2-yl]-2,5-diphenyltetrazolium bromide
MβCD: Methyl-β-cyclodextrin
NMR: Nuclear magnetic resonance
PGK: Phosphoglycerate kinase
PM: Plasma membrane
RCT: Reverse cholesterol pathway
ZA: Zaragozic acid
ΔFBS: Delipidated FBS

## References

[1] Chudzik B, Tracz IB, Czernel G, Fiolka MJ, Borsuk G, Gagos M (2013). Amphotericin B-copper(II) complex as a potential agent with higher antifungal activity against *Candida albicans*. Eur J Pharm Sci.

[2] Gruszecki WI, Gagos M, Kernen P (2002). Polyene antibiotic amphotericin B in monomolecular layers: spectrophotometric and scanning force microscopic analysis. FEBS Lett.

[3] Ibrahim F, Gershkovich P, Sivak O, Wasan EK, Wasan KM (2013). Pharmacokinetics and tissue distribution of amphotericin B following oral administration of three lipid-based formulations to rats. Drug Dev Ind Pharm.

[4] Gruszecki WI, Luchowski R, Gagos M, Arczewska M, Sarkar P, Herec M (2009). Molecular organization of antifungal antibiotic amphotericin B in lipid monolayers studied by means of fluorescence lifetime imaging microscopy. Biophys Chem.

[5] Neumann A, Baginski M, Winczewski S, Czub J (2013). The effect of sterols on amphotericin B self-aggregation in a lipid bilayer as revealed by free energy simulations. Biophys J.

[6] Kamiński DM, Czernel G, Murphy B, Runge B, Magnussen OM, Gagoś M (2014). Effect of cholesterol and ergosterol on the antibiotic amphotericin B interactions with dipalmitoylphosphatidylcholine monolayers: X-ray reflectivity study. Biochim Biophys Acta.

[7] Anderson TM, Clay MC, Cioffi AG, Diaz KA, Hisao GS, Tuttle MD (2014). Amphotericin forms an extramembranous and fungicidal sterol sponge. Nat Chem Biol.

[8] Matsumori N, Tahara K, Yamamoto H, Morooka A, Doi M, Oishi T (2009). Direct interaction between amphotericin B and ergosterol in lipid bilayers as revealed by 2H NMR spectroscopy. J Am Chem Soc.

[9] Foglia F, Fragneto G, Clifton LA, Lawrence MJ, Barlow DJ (2014). Interaction of amphotericin B with lipid monolayers. Langmuir.

[10] Engelman DM (2005). Membranes are more mosaic than fluid. Nature.

[11] He HT, Marguet D (2011). Detecting nanodomains in living cell membrane by fluorescence correlation spectroscopy. Annu Rev Phys Chem.

[12] Marguet D, Lenne P-F, Rigneault H, He H-T (2006). Dynamics in the plasma membrane: how to combine fluidity and order. EMBO J.

[13] Nicolson GL (2014). The fluid-mosaic model of membrane structure: still relevant to understanding the structure, function and dynamics of biological membranes after more than 40 years. Biochim Biophys Acta.

[14] Rossy J, Ma Y, Gaus K (2014). The organisation of the cell membrane: do proteins rule lipids?. Curr Opin Chem Biol.

[15] Attie AD (2007). ABCA1: at the nexus of cholesterol, HDL and atherosclerosis. Trends Biochem Sci.

[16] Phillips MC (2014). Molecular mechanisms of cellular cholesterol efflux. J Biol Chem.

[17] Phillips MC (2018). Is ABCA1 a lipid transfer protein?. J Lipid Res.

[18] Rosenson RS, Brewer HB, Davidson WS, Fayad ZA, Fuster V, Goldstein J (2012). Cholesterol efflux and atheroprotection: advancing the concept of reverse cholesterol transport. Circulation.

[19] Zarubica A, Trompier D, Chimini G (2007). ABCA1, from pathology to membrane function. Pflugers Arch.

[20] Quazi F, Molday RS (2013). Differential phospholipid substrates and directional transport by ATP-binding cassette proteins ABCA1, ABCA7, and ABCA4 and disease-causing mutants. J Biol Chem.

[21] Brooks-Wilson A, Marcil M, Clee SM, Zhang LH, Roomp K, van Dam M (1999). Mutations in ABC1 in Tangier disease and familial high-density lipoprotein deficiency. Nat Genet.

[22] Qian H, Zhao X, Cao P, Lei J, Yan N, Gong X (2017). Structure of the human lipid exporter ABCA1. Cell.

[23] Hamon Y, Broccardo C, Chambenoit O, Luciani MF, Toti F, Chaslin S (2000). ABC1 promotes engulfment of apoptotic cells and transbilayer redistribution of phosphatidylserine. Nat Cell Biol.

[24] Marguet D, Luciani MF, Moynault A, Williamson P, Chimini G (1999). Engulfment of apoptotic cells involves the redistribution of membrane phosphatidylserine on phagocyte and prey. Nat Cell Biol.

[25] Zarubica A, Plazzo AP, Stöckl M, Trombik T, Hamon Y, Müller P (2009). Functional implications of the influence of ABCA1 on lipid microenvironment at the plasma membrane: a biophysical study. FASEB J.

[26] Sorci-Thomas MG, Owen JS, Fulp B, Bhat S, Zhu X, Parks JS (2012). Nascent high density lipoproteins formed by ABCA1 resemble lipid rafts and are structurally organized by three apoA-I monomers. J Lipid Res.

[27] Guo D, Reinitz F, Youssef M, Hong C, Nathanson D, Akhavan D (2011). An LXR agonist promotes glioblastoma cell death through inhibition of an EGFR/AKT/SREBP-1/LDLR-dependent pathway. Cancer Discov.

[28] Kaneko T, Kanno C, Ichikawa-Tomikawa N, Kashiwagi K, Yaginuma N, Ohkoshi C (2015). Liver X receptor reduces proliferation of human oral cancer cells by promoting cholesterol efflux via up-regulation of ABCA1 expression. Oncotarget.

[29] Qin JY, Zhang L, Clift KL, Hulur I, Xiang AP, Ren BZ (2010). Systematic comparison of constitutive promoters and the doxycycline-inducible promoter. PLoS One.

[30] Sladitschek HL, Neveu PA (2015). MXS-chaining: a highly efficient cloning platform for imaging and flow cytometry approaches in mammalian systems. PLoS One.

[31] Churchward MA, Rogasevskaia T, Höfgen J, Bau J, Coorssen JR (2005). Cholesterol facilitates the native mechanism of Ca^2+^-triggered membrane fusion. J Cell Sci.

[32] Yang A, Gelissen IC (2017). ABC-transporter mediated sterol export from cells using radiolabeled sterols. Methods Mol Biol.

[33] Wójtowicz K, Gruszecki WI, Walicka M, Barwicz J (1998). Effect of amphotericin B on dipalmitoylphosphatidylcholine membranes: calorimetry, ultrasound absorption and monolayer technique studies. Biochim Biophys Acta Biomembr.

[34] Grela E, Wieczór M, Luchowski R, Zielinska J, Barzycka A, Grudzinski W (2018). Mechanism of binding of antifungal antibiotic amphotericin b to lipid membranes: an insight from combined single-membrane imaging, microspectroscopy, and molecular dynamics. Mol Pharm.

[35] Wang X, Collins HL, Ranalletta M, Fuki IV, Billheimer JT, Rothblat GH (2007). Macrophage ABCA1 and ABCG1, but not SR-BI, promote macrophage reverse cholesterol transport in vivo. J Clin Invest.

[36] Hong C, Walczak R, Dhamko H, Bradley MN, Marathe C, Boyadjian R (2011). Constitutive activation of LXR in macrophages regulates metabolic and inflammatory gene expression: identification of ARL7 as a direct target. J Lipid Res.

[37] Ito A, Hong C, Rong X, Zhu X, Tarling EJ, Hedde PN (2015). LXRs link metabolism to inflammation through Abca1-dependent regulation of membrane composition and TLR signaling. Elife.

[38] Wojcik AJ, Skaflen MD, Srinivasan S, Hedrick CC (2008). A critical role for ABCG1 in macrophage inflammation and lung homeostasis. J Immunol.

[39] Trompier D, Alibert M, Davanture S, Hamon Y, Pierres M, Chimini G (2006). Transition from dimers to higher oligomeric forms occurs during the ATPase cycle of the ABCA1 transporter. J Biol Chem.

[40] Favari E, Zanotti I, Zimetti F, Ronda N, Bernini F, Rothblat GH (2004). Probucol inhibits ABCA1-mediated cellular lipid efflux. Arterioscler Thromb Vasc Biol.

[41] Wu CA, Tsujita M, Hayashi M, Yokoyama S (2004). Probucol inactivates ABCA1 in the plasma membrane with respect to its mediation of apolipoprotein binding and high density lipoprotein assembly and to its proteolytic degradation. J Biol Chem.

[42] Yamamoto S, Tanigawa H, Li X, Komaru Y, Billheimer JT, Rader DJ (2011). Pharmacologic suppression of hepatic ATP-binding cassette transporter 1 activity in mice reduces high-density lipoprotein cholesterol levels but promotes reverse cholesterol transport. Circulation.

[43] Grudzinski W, Sagan J, Welc R, Luchowski R, Gruszecki WI (2016). Molecular organization, localization and orientation of antifungal antibiotic amphotericin B in a single lipid bilayer. Sci Rep.

[44] Starzyk J, Gruszecki M, Tutaj K, Luchowski R, Szlazak R, Wasko P (2014). Self-association of amphotericin b: spontaneous formation of molecular structures responsible for the toxic side effects of the antibiotic. J Phys Chem B.

[45] Bergstrom JD, Kurtz MM, Rew DJ, Amend AM, Karkas JD, Bostedor RG (1993). Zaragozic acids: a family of fungal metabolites that are picomolar competitive inhibitors of squalene synthase (cholesterol synthesis inhibitors/fungal metabolites). Proc Natl Acad Sci USA.

[46] Lasserre R, Guo X-J, Conchonaud F, Hamon Y, Hawchar O, Bernard A-M (2008). Raft nanodomains contribute to Akt/PKB plasma membrane recruitment and activation. Nat Chem Biol.

[47] Hamill RJ (2013). Amphotericin B formulations: a comparative review of efficacy and toxicity. Drugs.

[48] Torrado JJ, Espada R, Ballesteros MP, Torrado-Santiago S (2008). Amphotericin B formulations and drug targeting. J Pharm Sci.

[49] Kontoyiannis DP, Marr KA, Park BJ, Alexander BD, Anaissie EJ, Walsh TJ (2010). Prospective surveillance for IFI in HSCT recipients. CID.

[50] Duong PT, Nickel M, Nguyen D, Dhanasekaran P, Saito H, Rothblat GH (2007). Mechanism of ATP-binding cassette transporter A1-mediated cellular lipid efflux to apolipoprotein A-I and formation of high density lipoprotein particles. J Biol Chem.

[51] Reboul E, Dyka FM, Quazi F, Molday RS (2013). Cholesterol transport via ABCA1: new insights from solid-phase binding assay. Biochimie.

[52] Nagata KO, Nakada C, Kasai RS, Kusumi A, Ueda K (2013). ABCA1 dimer-monomer interconversion during HDL generation revealed by single-molecule imaging. Proc Natl Acad Sci USA.

[53] Ishigami M, Ogasawara F, Nagao K, Hashimoto H, Kimura Y, Kioka N (2018). Temporary sequestration of cholesterol and phosphatidylcholine within extracellular domains of ABCA1 during nascent HDL generation. Sci Rep.

[54] Kennedy MA, Barrera GC, Nakamura K, Baldán Á, Tarr P, Fishbein MC (2005). ABCG1 has a critical role in mediating cholesterol efflux to HDL and preventing cellular lipid accumulation. Cell Metab.

[55] Purkait B, Kumar A, Nandi N, Hasan Sardar A, Das S, Kumar S (2012). Mechanism of amphotericin B resistance in clinical isolates of *Leishmania donovani*. Antimicrob Agents Chemother.

[56] Schinkel AH, Jonker JW (2012). Mammalian drug efflux transporters of the ATP binding cassette (ABC) family: an overview. Adv Drug Deliv Rev.

[57] Vtorushin SV, Khristenko KY, Zavyalova MV, Perelmuter VM, Litviakov NV, Denisov EV (2014). The phenomenon of multi-drug resistance in the treatment of malignant tumors. Exp Oncol.

[58] Huang W, Zhang Z, Han X, Tang J, Wang J, Dong S (2002). Ion channel behavior of amphotericin B in sterol-free and cholesterol- or ergosterol-containing supported phosphatidylcholine bilayer model membranes investigated by electrochemistry and spectroscopy. Biophys J.

[59] Gray KC, Palacios DS, Dailey I, Endo MM, Uno BE, Wilcock BC (2012). Amphotericin primarily kills yeast by simply binding ergosterol. Proc Natl Acad Sci.

[60] Grela E, Piet M, Luchowski R, Grudzinski W, Paduch R, Gruszecki WI (2018). Imaging of human cells exposed to an antifungal antibiotic amphotericin B reveals the mechanisms associated with the drug toxicity and cell defence. Sci Rep.

[61] Muraglia KA, Chorghade RS, Kim BR, Tang XX, Shah VS, Grillo AS (2019). Small-molecule ion channels increase host defences in cystic fibrosis airway epithelia. Nature.

[62] Vaughan AM, Oram JF (2003). ABCA1 redistributes membrane cholesterol independent of apolipoprotein interactions. J Lipid Res.

[63] Bocer T, Zarubica A, Roussel A, Flis K, Trombik T, Goffeau A (2012). The mammalian ABC transporter ABCA1 induces lipid-dependent drug sensitivity in yeast. Biochim Biophys Acta.

[64] Zhang L (2002). Response of gene expression in *Saccharomyces cerevisiae* to amphotericin B and nystatin measured by microarrays. J Antimicrob Chemother.

[65] Alvarez FJ, Douglas LM, Konopka JB (2007). Sterol-rich plasma membrane domains in fungi. Eukaryot Cell.

[66] Baginski M, Resat H, Borowski E (2002). Comparative molecular dynamics simulations of amphotericin B–cholesterol/ergosterol membrane channels. Biochim Biophys Acta Biomembr.

[67] Ren B, Dai H-Q, Pei G, Tong Y-J, Zhuo Y, Yang N (2014). ABC transporters coupled with the elevated ergosterol contents contribute to the azole resistance and amphotericin B susceptibility. Appl Microbiol Biotechnol.

[68] Naik SU, Wang X, Da Silva JS, Jaye M, Macphee CH, Muredach (2006). Pharmacological activation of liver X receptors promotes reverse cholesterol transport in vivo. Circulation.

[69] Kobayashi M, Gouda K, Chisaki I, Ochiai M, Itagaki S, Iseki K (2011). Molecular and cellular pharmacology regulation mechanism of ABCA1 expression by statins in hepatocytes. Eur J Pharmacol.

