# Supplementary material for: ABCA1 transporter reduces amphotericin B cytotoxicity in mammalian cells
Source: Cell Mol Life Sci. 2019 May 27;76(24):4979–94. doi: 10.1007/s00018-019-03154-w (PMC6881254; doi:10.1007/s00018-019-03154-w)
Supplement: Supplementary file 1 — Supplementary material 1 (DOCX 4667 kb) [file 18_2019_3154_MOESM1_ESM.docx]

**ABCA1 transporter reduces amphotericin B cytotoxicity in mammalian cells**

Wu, A.^1^, Grela, E.^2^, Wójtowicz, K.^1^, Filipczak, N.^1^, Hamon, Y.^3^, Luchowski, R.^2^, Grudziński, W.^2^, Raducka-Jaszul, O.^1^, Gagoś, M.^4^, Szczepaniak, A.^1^, Chimini, G.^3^, Gruszecki, W.I.^2^, Trombik, T.^1*^

^1^ Faculty of Biotechnology, University of Wroclaw, 50-383 Wroclaw, Poland

^2^ Department of Biophysics, Institute of Physics, Maria Curie-Sklodowska University, 20-031 Lublin, Poland.

^3^ Aix Marseille University, CNRS, INSERM, CIML, Marseille, France

^4^ Department of Cell Biology, Maria Curie-Skłodowska University, 20-033 Lublin, Poland

^*^correspondence to: [tomasz.trombik@uwr.edu.pl](mailto:tomasz.trombik@uwr.edu.pl); telephone: +48 71 375 62 43

**Supplementary figures**


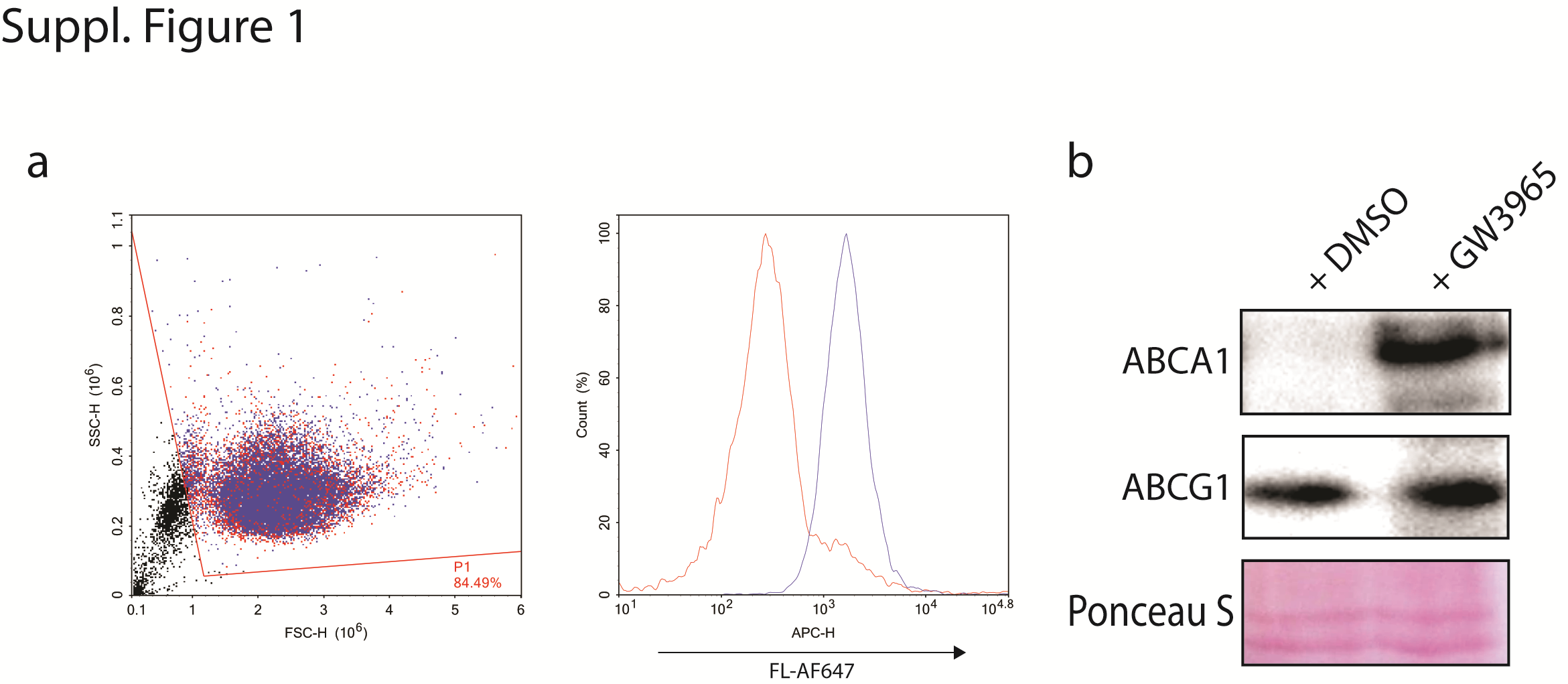


**Supplementary Figure 1. ABCA1 expression in Raw 264.7 macrophages.**

(**a**) Flow cytometry analysis of ABCA1 expression in Raw 264.7 macrophages using anti-ABCA1 antibody (clone 5A1-1422) directly coupled to Alexa Fluor 647. Mock cells (DMSO) in red, GW3965 treated cells in blue. Left panel shows a scatter plot of cell populations in analyzed gate (red) and right panel shows a histogram of Alexa Fluor 647 fluorescence in APC channel (FL-AF647). (**b**) Western blot analysis of cell extracts from Raw 264.7 cells treated or not with 1 µM GW3965. The presence of protein was revealed with either anti-ABCA1 (clone 3A1-893.1) or anti-ABCG1 antibody. Upper panel shows detection of signal from ABCA1 protein, middle panel from ABCG1 protein and bottom panel shows a Ponceau Red S stained membrane as a loading control.


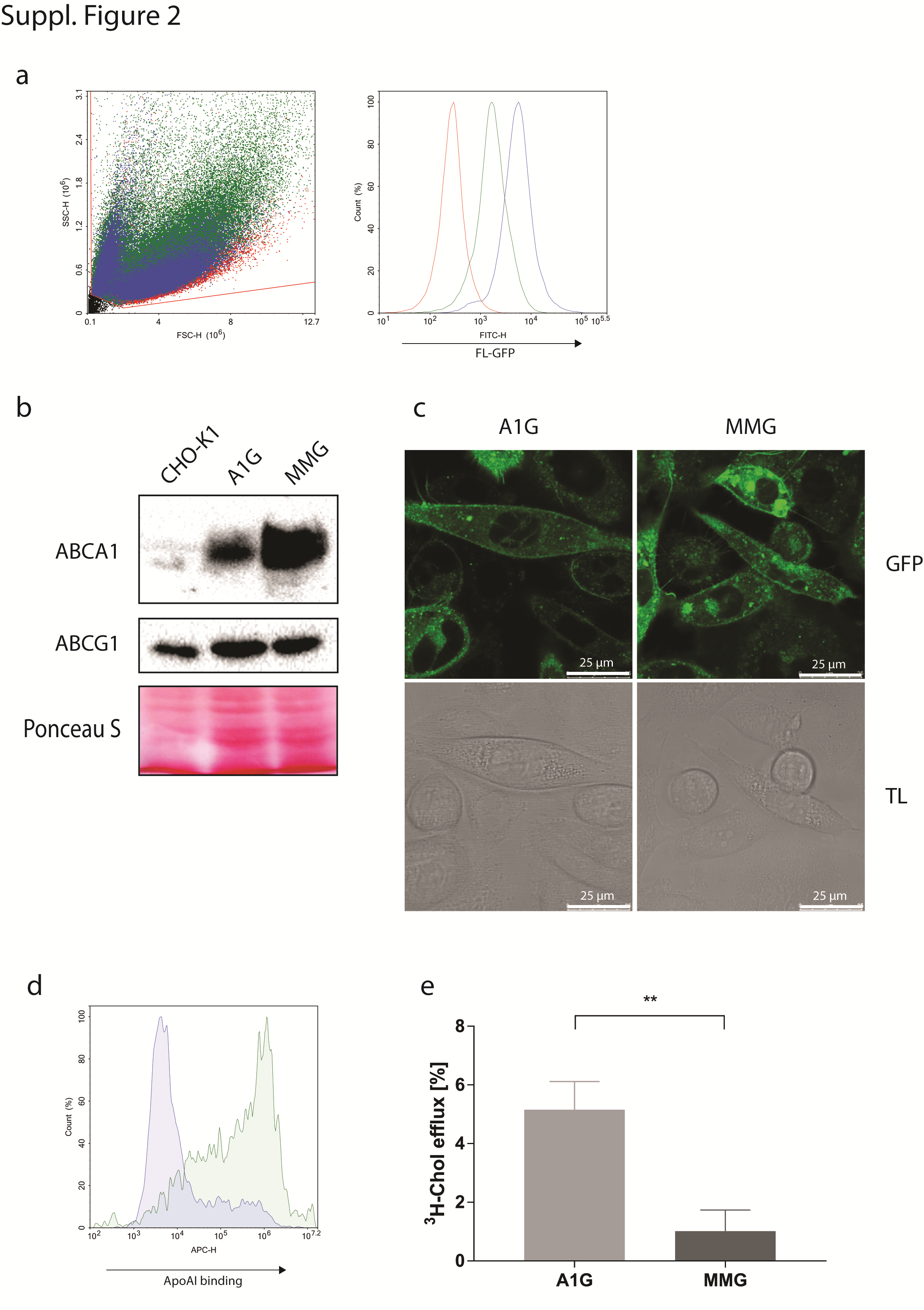


**Supplementary Figure 2.** **CHO-K1 cell lines stably expressing ABCA1.**

**(a**) Flow cytometry analysis of wild-type CHO-K1 (red), A1G (green) and MMG (blue) cells. Left panel shows a scatter plot of cell populations in analyzed gate (red) and right panel shows a histogram of GFP cell fluorescence (FL-GFP in FITC-H channel). (**b**) Western blot analysis of cell extracts from CHO-K1, A1G and MMG cells. The presence of protein was revealed with either anti-ABCA1 (clone 3A1-893.1) or anti-ABCG1 antibody. Upper panel shows detection of signal from ABCA1 protein, middle panel signal from ABCG1 protein and bottom panel shows a Ponceau Red S stained membrane as a loading control. (**c**) Confocal images of living A1G (left panels) and MMG (right panels) cells. Upper panels show GFP channel and bottom panels show transmitted light channel (TL). Scale bar corresponds to 25 µm. (**d**) Analysis of ApoAI coupled with Alexa Fluor 647 (ApoAI-AF647) binding. The panel shows a histogram of cell fluorescence in APC channel showing ApoAI binding (ApoAI-AF647 in APC channel). A1G cells in green and MMG cells in blue. (e) Graph representing [^3^H]-cholesterol efflux to ApoAI as an acceptor in A1G and MMG cells expressed in percent of efflux (y-axis). The significance of difference in efflux between A1G vs. MMG was validated with two-tailed t-test at the confidence level of 95% (**p<0.005). Error bars correspond to SD. The data were obtained in single experiment, in triplicate.


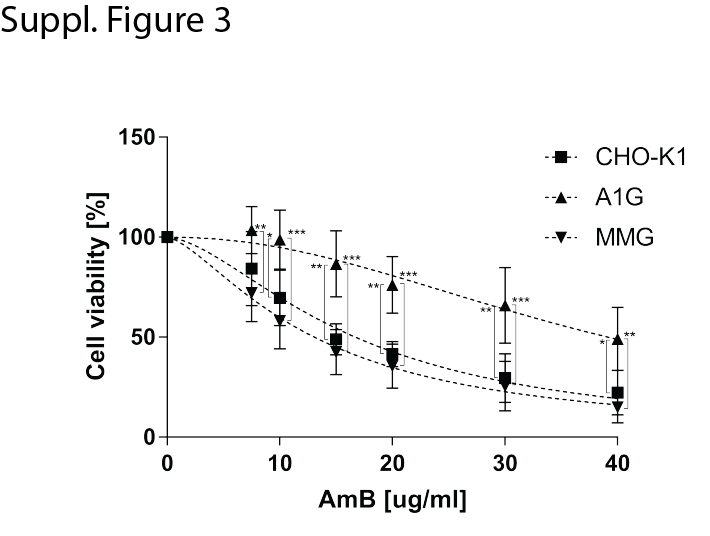


**Supplementary Figure 3.** **Comparison of cell viability after AmB treatment between CHO-K1 wild-type cells, A1G and MMG cell lines.**

Graph representing a nonlinear fit (dashed line) of cell viability as a function of decreased AmB concentrations assessed by MTT assay in CHO-K1, A1G and MMG cells. Axes represent AmB concentration in µg/ml (x-axis) and cell viability in percent of control (y-axis). Two-way ANOVA (alpha 0.05) with Sidak’s multiple comparison test was used to assess the significance of differences (***p<0.001, **p<0.01, *p<0.1). Error bars correspond to SD. The data were obtained in three independent experiments, each in triplicate.


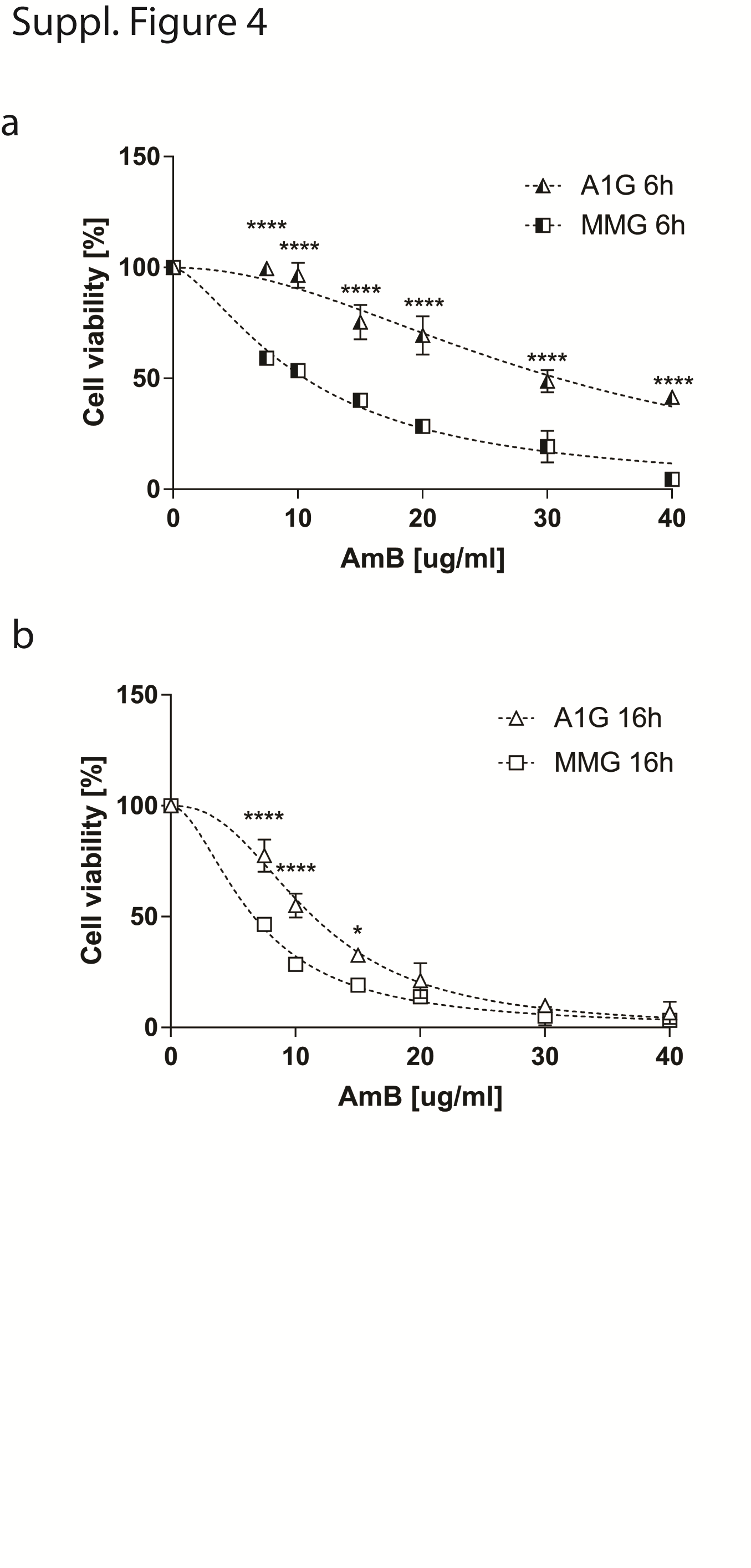


**Supplementary Figure 4.** **Comparison of A1G and MMG cells viability after AmB treatment for 6h
and 16h.**

Graph representing a nonlinear fit (dashed line) of cell viability as a function of decreased AmB concentrations assessed by MTT assay in A1G and MMG cells treated with AmB for 6h (a) and 16h (b). Axes represent AmB concentration in µg/ml (x-axis) and cell viability in percent of control (y-axis). Two-way ANOVA (alpha 0.05) with Sidak’s multiple comparison test was used to assess the significance of differences (****p<0.0001, ***p<0.001, **p<0.01, *p<0.1). Error bars correspond to SD. The data were obtained in two independent experiments, each in triplicate.


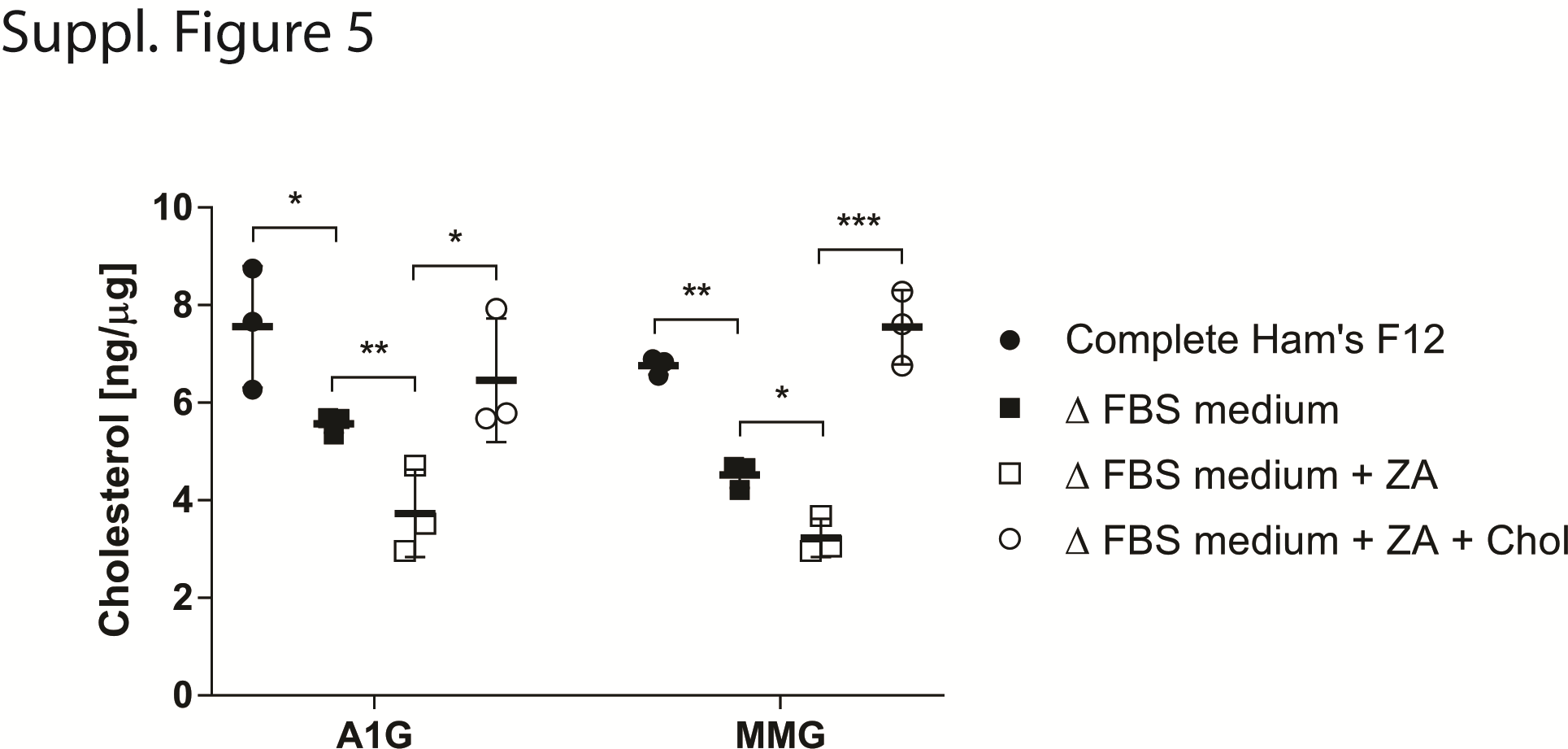


**Supplementary Figure 5. Cholesterol content in ABCA1-expressing cell lines.**

Graph representing a total cholesterol content of A1G and MMG cells expressed as ng of cholesterol per µg of protein. The cells were cultured in identical conditions as for cytotoxicity experiments in complete Ham’s F12 medium, ΔFBS medium, ΔFBS medium with zaragozic acid (ΔFBS medium + ZA) and ΔFBS medium with ZA and reloaded with cholesterol (ΔFBS medium + ZA + Chol). Two-way ANOVA (alpha 0.05) with Sidak’s multiple comparison test was used to assess the significance of differences (***p<0.001, **p<0.01, *p<0.1). The comparisons were made by groups of two. Error bars correspond to SD. The data were obtained in three independent experiments.


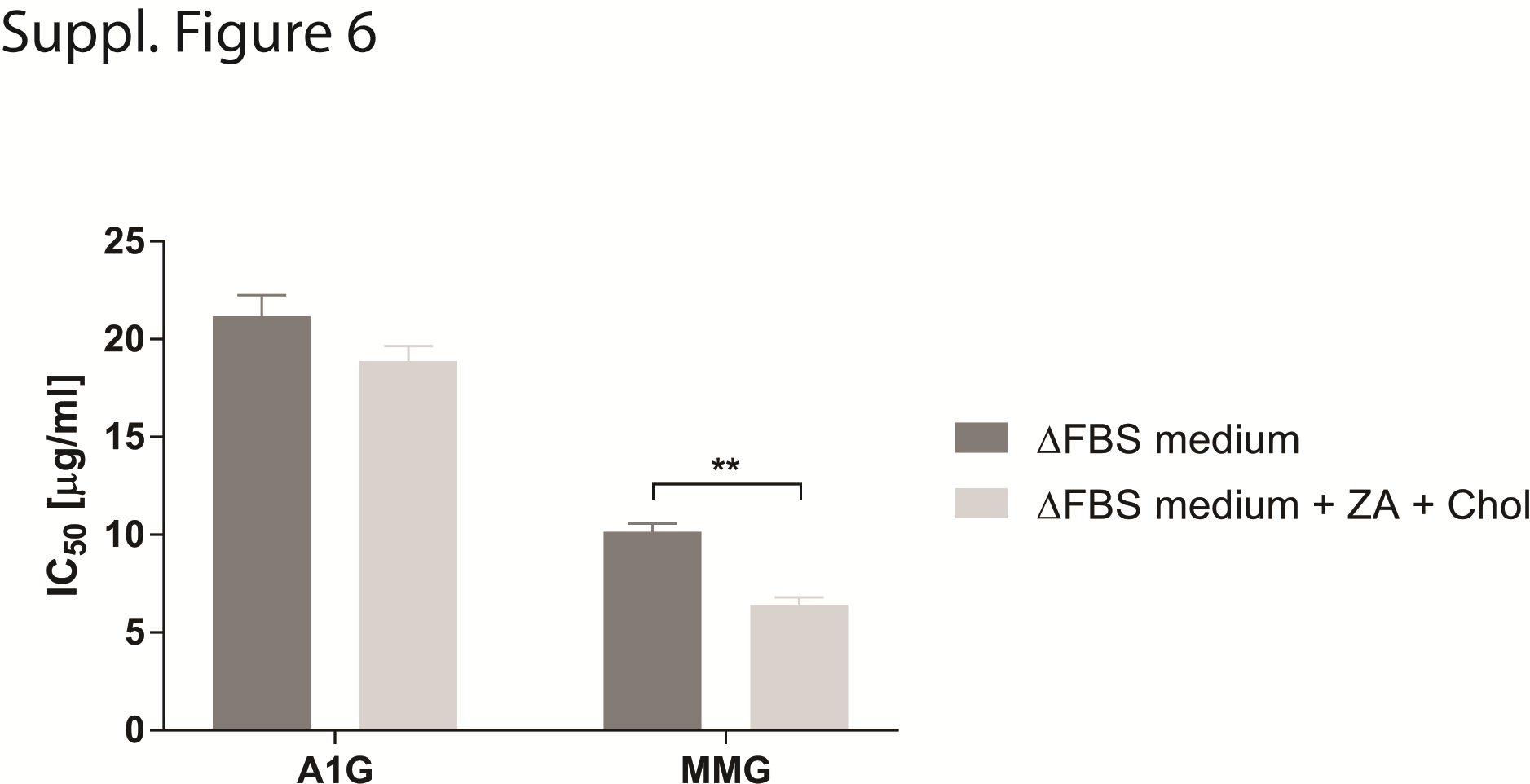


**Supplementary Figure 6. Comparison of IC_50_ for AmB after cholesterol reload.**

Graph representing a comparison of IC_50_ values for AmB from A1G and MMG cells cultured either in ΔFBS medium or in ΔFBS medium + ZA + Chol. Two-way ANOVA (alpha 0.05) with Sidak’s multiple comparison test was used to assess the significance of differences (**p<0.01). Error bars correspond to SD.
